# Comparative Assessment of Habitat Suitability and Niche Overlap of Three *Cytospora* Species in China

**DOI:** 10.3390/jof10010038

**Published:** 2024-01-03

**Authors:** Chengcai Yan, Haiting Hao, Shuaishuai Sha, Zhe Wang, Lili Huang, Zhensheng Kang, Lan Wang, Hongzu Feng

**Affiliations:** 1 College of Life Science and Technology, Tarim University, Alar 843300, China; yancc119@126.com; 2 Key Laboratory of Integrated Pest Management (IPM) of Xinjiang Production and Construction Corps in Southern Xinjiang, Tarim University, Alar 843300, China; m18919046163@163.com (H.H.); huanglili@nwafu.edu.cn (L.H.); kangzs@nwsuaf.edu.cn (Z.K.); 3 The National and Local Joint Engineering Laboratory of High Efficiency and Superior-Quality Cultivation and Fruit Deep Processing Technology of Characteristic Fruit Trees in Southern Xinjiang, Tarim University, Alar 843300, China; 4 College of Modern Agriculture, Kashgar University, Kashgar 844006, China; 5 State Key Laboratory of Crop Stress Biology for Arid Areas, Northwest A&F University, Xianyang 712100, China

**Keywords:** climate warming, fungal pathogens, habitat shifts, niche overlap, species distribution model

## Abstract

The plant pathogenic fungus *Cytospora* is notoriously known for causing woody plant canker diseases, resulting in substantial economic losses to biological forests and fruit trees worldwide. Despite their strong negative ecological impact, the existing and prospective distribution patterns of these plant pathogens in China, according to climate change, have received little attention. In this study, we chose three widely dispersed and seriously damaging species, namely, *Cytospora chrysosperma*, *Cytospora mali*, and *Cytospora nivea*, which are the most common species that damage the *Juglans regia*, *Malus domestica*, *Eucalyptus*, *Pyrus sinkiangensis*, *Populus* spp., and *Salix* spp. in China. We utilized ecological niche modeling to forecast their regional distribution in China under four climate change scenarios (present, SSP 126, SSP 370, and SSP 585). The results show that temperature-related climate factors limit the current distribution ranges of the three species. Currently, the three studied species are highly suitable for northeast, northwest, north, and southwest China. Under future climate scenarios, the distribution ranges of the three species are projected to increase, and the centers of the adequate distribution areas of the three species are expected to shift to high-latitude regions. The three species coexist in China, primarily in the northwest and north regions. The ecological niches of *C. chrysosperma* and *C. nivea* are more similar. The distribution range of *C. mali* can reach the warmer and wetter eastern region, whereas *C. chrysosperma* and *C. nivea* are primarily found in drought-prone areas with little rainfall. Our findings can help farmers and planners develop methods to avoid the spread of *Cytospora* spp. and calculate the costs of applying pesticides to reduce contamination and boost yields.

## 1. Introduction

Plant pathogens have significant impacts on plant output all around the world [1]. Climate change can directly lead to plant production losses through agricultural meteorological disasters or indirectly through the impacts of plant pathogens [2]. Many studies have found that climate change is a major driver of plant pathogen spatial pattern changes and may improve plant pathogen overwintering survivability [3,4]. The distribution range of many plant pathogens is expected to continue shifting toward high-latitude and high-altitude locations [5,6]. Thus, understanding the spatial distribution patterns, population diversity, and compositions of plant pathogens affected by climate is critical for reducing plant disease losses.

Members of the *Cytospora* genus are found worldwide and are frequently considered endophytes, saprobes, or phytopathogens that can infect many hosts [7,8]. *Cytospora* species are mostly connected with woody plant canker diseases such as plant infections. Other diseases, such as Chinese jujube root rot and pomegranate collar rot, have also been observed [9,10]. Wounds caused by biotic (insects and birds) and abiotic (wind, drought, frost, rain, and hail) stress are the primary ways that *Cytospora* species infect host plants [11,12,13], indicating that harsh circumstances are always favorable for these taxa in China [14]. The genus *Cytospora* has 110 species, 31 of which have been identified in China [15]. Among the numerous pathogens attacking important economic woody plants, such as *Juglans regia* [16], *Malus domestica* [17,18], *Eucalyptus* [7], *Pyrus sinkiangensis* [17,18], *Populus* spp. [19,20], and *Salix* spp. [13], *Cytospora chrysosperma*, *C. nivea*, and *C. mali* are the most common species and cause severe economic and ecological losses, especially in China [8,13,21]. Their occurrence, development, distribution, and prevalence are vital to the long-term development of China’s forestry industry in the northeast, northwest, north, and southwest [8,13].

Understanding the basic biological features of these three species may help us better appreciate their dangers and the need to anticipate future suitable areas. The first species is *C. chrysosperma*, which is thought to be the primary cause of *Salicaceae* canker disease, raising quarantine concerns, particularly in China [8,22]. The species is a typical *Cytospora* species with a wide host range across Europe, Asia, Africa, Australia, and America [7,14,23]. The optimal growth temperature of *C. chrysosperma* is 28 °C [24]. The second species is *C. mali*; like other *Cytospora* species, it is typically known as an opportunistic pathogen occurring within a relatively narrow host range and mainly attacks economically important fruit crops, such as *Malus domestica* and *Pyrus sinkiangensis* [15,25] in China and Japan. *C. mali* is one of the few well-studied critical pathogens in the genus *Cytospora* concerning its biology, infection, and populations. Previous research has shown that *C. mali* can survive in the xylem for five years [26] and that the conidia can maintain their germination potential for 16 days at −15 °C, indicating that the species has strong environmental adaptability [27,28]. The optimal growth temperature of *C. mali* is 25 °C [24]. The third species is *C. nivea*, which, like *C. chrysosperma*, is the second most common pathogenic fungus that mainly harms the host of the *Salicaceae* family in Asia, Europe, and South America [13,29,30]. Meanwhile, it can also harm *Juglans regia* [16], *Malus domestica* [18], and *Pyrus sinkiangensis* [17]. The optimal growth temperature of *C. nivea* is 25 °C [24]. From the above, we speculate that the distribution ranges, niche overlap, and climatic preferences may differ between these species, but their geographical distributions and determinants have not yet been addressed. Thus, additional disease management strategies are needed.

Global temperature change is widely acknowledged to significantly impact species distribution [6,31,32] and may increase fungal infections [6,33]. However, few studies [5,34,35] have explored the dispersion of fungi on large spatial and temporal scales due to their distinctive life cycles and development patterns. Determining the potential geographic range of the *Cytospora* canker is crucial for field management decision making and surveillance. GIS (Geographic information systems) and SDM (spatial data management) present a possible solution to this problem [3]. They will expand across more species, geographic regions, and research topics [5,36]. 

Currently, niche models like the Maxent (Maximum Entropy) model [37], ENFA (Ecological Niche Factor Analysis) model [38], CLIMEX (Climate Change Experiment) [39], and GARP (Genetic Algorithm for Rule-set Production) model [40] and GLMs (Generalized Linear Models) [41] are widely used to simulate and predict the appropriate areas of fungal diseases [5,42,43,44]. The Maxent modeling approach is widely utilized among these models due to its superior predictive performance [5,45,46]. It is effective even when few occurrence records are known and the association between climatic and environmental elements is unpredictable [6,44,47]. Therefore, we used the Maxent model to analyze the potential distribution of the chosen species.

Given that *C. chrysosperma*, *C. mali*, and *C. nivea* have been linked to woody plant canker diseases, elucidating their ecological and physiological characteristics, such as distribution ranges, habitat shifts, niche overlap, and climatic preferences, would aid in disease prediction and the consideration of their controls. Therefore, the following problems are addressed in this study: (1) determining the key environmental parameters impacting *C. chrysosperma*, *C. mali*, and *C. nivea* distributions; (2) calculating the core distribution for each species to estimate their appropriate areas under present and future climatic scenarios and evaluating their migration propensity; and (3) computing and comparing species’ niche overlap. We hope our findings will be valuable to policymakers in developing measures to combat *Cytospora* canker disease. 

## 2. Materials and Methods

### 2.1. Occurrence Records

**Figure 1:**
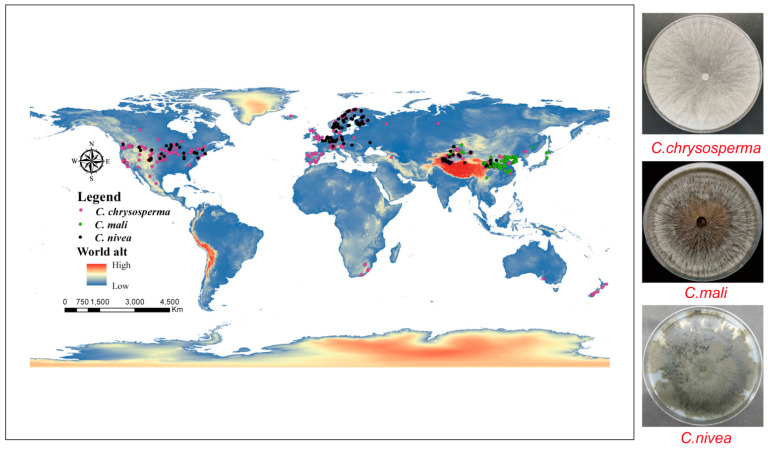
Global Occurrence records of three studied species. The pink, green and black dots represent *C. chrysosperma*, *C. mali* and *C. nivea*, respectively.

All georeferenced occurrence records in our study were obtained from three different sources: (1) GBIF (The Global Biodiversity Information Facility) (https://www.gbif.org/); (2) relevant articles from the China National Knowledge Infrastructure (CNKI) (https://www.cnki.net/), Web of Science (WOS) (https://www.webofscience.com/), and Google Scholar (https://scholar.google.com.hk); and (3) GPS, which was used to gather 135 *C. chrysosperma*, 34 *C. mali*, and 25 *C. nivea* occurrence points during fieldwork in the Xinjiang Uygur Autonomous Region, China in 2019 and 2023. By ensuring that no two occurrence data points were inside the same raster (~5 km^2^) [6,48], ENMTools (https://github.com/danlwarren/ENMTools) was utilized to prevent spatial autocorrelation from impairing the model’s performance [48,49]. Ultimately, 374 global occurrence data points for *C. chrysosperma*, 164 for *C. mali*, and 166 for *C. nivea* were retained by our study (Figure 1 and Supplementary Table S1).

### 2.2. Environmental Factor Variables

Numerous factors, such as the habitat’s climatic suitability, topography, land cover, and dispersal history, are probably responsible for the spread of numerous fungi [5,21,50]. Since *C. chrysosperma*, *C. mali*, and *C. nivea* typically affect woody plants, we selected soil and vegetation conditions as limiting ecological variables in this study. Supplementary Table S2 displays the selected environmental variables, which include 19 bioclimatic factors, three topographical factors (aspect, slope, and altitude), one global land cover data point (~1 km), and 11 topsoil factors (0–30 cm) with a 2.5 arc-min spatial resolution. These variables were obtained from the World Climate website (http://www.worldclim.org), the Harmonized World Soil Database (http://www.fao.org/soils-portal/), and the Global Maps website (http://globalmaps.github.io). Future climate data were based on BCC-CSM2-MR data in the Coupled Model Intercomparison Project Phase 6 (CMIP6), which is more appropriate for Asia, particularly China [51,52]. To predict the future distributions of *C. chrysosperma*, *C. mali*, and *C. nivea* in 2030, 2050, 2070, and 2090, three independent socioeconomic models driven by CO_2_ and shared socioeconomic pathways (SSPs) 126, 370, and 585 were chosen in this study.

The species distribution models (SDMs) may overfit due to multicollinearity amongst bioclimatic variables [43]. To eliminate multivariate collinearity, we utilized ENMTools to evaluate the correlation coefficients of the bioclimatic variables. Finally, we kept several meaningful bioclimatic variables for each research species based on the correlation coefficient of the bioclimatic variables (|r| > 0.8) and the contribution of each bioclimatic variable (Supplementary Figure S1) [6,45,48].

### 2.3. Model Simulation, Optimization, and Evaluation

The regularization multiplier (RM) and feature classes (FCs) are the two most important parameters of the Maxent model, aside from occurrence records and associated bioclimatic variables. A suitable combination of these elements can raise the sensitivity of the model while lowering the risk of overfitting [45,53]. The Maxent model’s parameters were calibrated using the R 3.6.3 “Kuennm” package to determine the ideal model tuning settings for each research species. We examined 31 different FC combinations (L for linear, Q for quadratic, H for hinge, P for product, and T for threshold), and the RM varied progressively throughout a 0.1-unit interval from 0.1 to 4. With delta Akaike minimal information criterion (AICc) values as low as possible (delta AICc = 0 or <2) [54,55], the best Maxent models were identified. Finally, RMs of 0.4, 1.5, and 1.2 were chosen for *C. chrysosperma*, *C. mali*, and *C. nivea*, respectively, along with feature combinations of LQP, LQH, and QT (Table 1).

Then, we adjusted the parameters of the Maxent and completed the modeling of the studied species. Specifically, “Create response curves”, “Random seed”, “Do jackknife to measure variable importance”, “Write plot data”, “Write background predictions”, “Replicates 10”, “Replicated run type subsample”, “output file type ‘.asc’”, and “Output format logistic” were the parameters set by the Maxent model. Training data accounted for 75% of the distribution data, with testing data making up the remaining 25%. The default values for the other settings of the software were used. To assess the performance of the model, we used the values of the true skill statistic and area under the receiver operating characteristic (ROC) curve (AUC). AUC values vary from 0 to 1 [56]. A prediction is deemed bad if it is less than 0.5, acceptable if it is between 0.5 and 0.7, good if it is between 0.7 and 0.9, and outstanding if it is between 0.9 and 1 [6,57]. TSS was rated as failed, <0.4; good, 0.4–0.75; and excellent, >0.75 [58,59]. In addition, the efficacy of the model was confirmed in this study by contrasting the results with the field survey data.

### 2.4. Assessment of Current and Future Distribution Areas

ArcGIS 10.4.1 was used to visualize and further analyze the continuous habitat suitability maps created for each study species. Based on the maximum test sensitivity plus specificity (MTSPS) threshold [6,25,60], binary suitability maps (unsuitable and suitable areas) for each study species were constructed. We developed four climate suitability categories for the three species, unsuitable, low suitable, medium suitable, and high suitable, to interpret the prediction results more easily [6].

Changes in the distribution regions of species can be efficiently reflected by core distribution migration [5]. Therefore, we employed SDMtoolbox (version 2.4) [61] to derive the core distribution migration of three species under present and future concentration scenarios and explain changes in the distribution of the appropriate species areas more clearly. Core distribution migration results were obtained using SDMtoolbox (version 2.4) by importing the “.asc” files produced under various concentration scenarios predicted by the Maxent model into ArcGIS 10.4.1. After that, additional analysis was conducted on the direction and distance of the core distribution migration [61]. Additionally, we calculated the contraction and expansion of the potential range of *C. chrysosperma*, *C. mali*, and *C. nivea* in China with climate change using SDMtoolbox (version 2.4).

### 2.5. Niche Overlap Analysis

ENMTools (version 1.3) was used to compare the ecological niches of our research species to Maxent projections. Hellinger’s I and Schoener’s D values represented the degree of niche overlap. D values range from 0 (no overlap) to 1 (more than 0.6 indicates significant overlap) [48,62,63]. Additionally, we used ArcGIS 10.4.1 to identify the homologous distribution areas of the three species and mapped the overlapping areas of the species niches under various conditions [43,45].

## 3. Results

### 3.1. Model Performance and Variable Contribution

**Figure 2:**
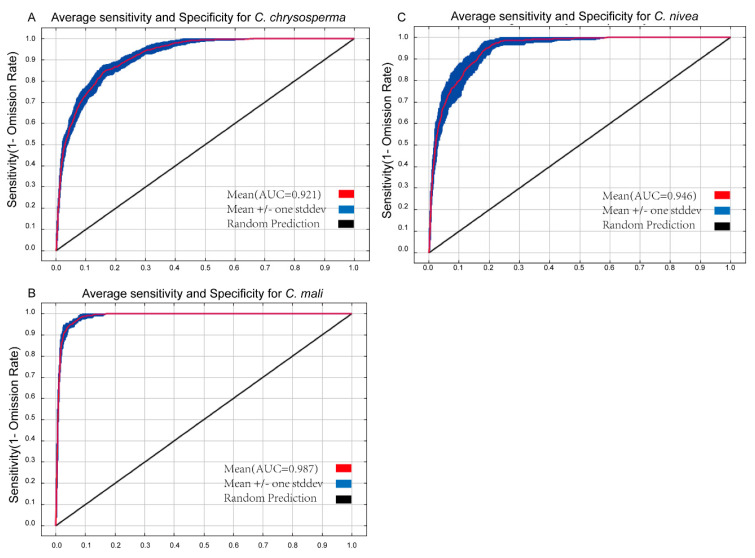
The receiver operating characteristic curve for target species. (**A**–**C**) represent *C. chrysosperma*, *C. mali* and *C. nivea*, respectively.

AUC and TSS values have frequently been employed to assess SDM model performance [52,64]. The greater the value, the stronger the relationship between bioclimatic factors and the expected distribution area, and the better the model works [65,66]. The average AUC values for 10 repetitions for *C. chrysosperma*, *C. mali*, and *C. nivea* were 0.921, 0.987, and 0.946 (Figure 2), respectively. Their TSS values were 0.797, 0.898, and 0.802, all exceeding 0.79 (Table 1 and Supplementary Figure S2), indicating that the optimized Maxent model predicted the potential distribution well. 

Based on detailed jackknife testing and a percent contribution study, the main influencing factors for the geographical distribution of the three research species are different (Supplementary Figure S3). The most important bioclimatic variable in the predictions for *C. chrysosperma* was the annual mean temperature (Bio 1, 64.4%). Other important bioclimatic variables included land cover (8.6%), mean diurnal range (Bio 2, 7.9%), seasonality of precipitation (Bio 15, 7.9%), and topsoil gravel content (T-grave, 7.6%), with a total contribution rate of up to 96.4% (Supplementary Table S3). The mean temperature of the driest quarter (Bio 9, 35.4%) had the highest mean contribution to the *C. mali* models, followed by precipitation of the coldest quarter (Bio 19, 18.8%), precipitation of the warmest quarter (Bio 18, 11.3%), land cover (11%), and temperature seasonality (Bio 4, 9.5%), with a total contribution rate of up to 86% (Supplemental Table S3). The most significant environmental variable that affected the AUC of the models for *C. nivea* was the mean temperature of the coldest quarter (Bio 11, 67.7%). This was followed by the precipitation of the wettest month (Bio 13, 9.2%), the mean diurnal range (Bio 2, 7.2%), and the topsoil TEB (5.4%), with a total contribution rate of up to 89.5% (Supplementary Table S3).

For *C. chrysosperma*, the optimal ranges of the mean diurnal range (Bio 2), annual mean temperature (Bio 1), land cover, and precipitation seasonality (Bio 15) were >1.9 °C, −2.76–17.19 °C, >3.18, and 0–120.14 mm, respectively (Supplementary Figure S3A and Supplementary Table S4). For *C. mali*, the optimal ranges of the precipitation of the coldest quarter (Bio 19), mean temperature of the driest quarter (Bio 9), precipitation of the warmest quarter (Bio 18), land cover, and temperature seasonality (Bio 4) were >0–46.55 mm, >−10.46–9.17 °C, 8.57–927.25 mm, and 7.08–19.23, respectively (Supplementary Figure S3B and Supplementary Table S4). For *C. nivea*, the optimal ranges of the precipitation of the wettest month (Bio 13), mean temperature of the coldest quarter (Bio 11), and T-teb were 0–154.09 mm, >−13.64–4.75 °C, and 0–46.58, respectively (Supplementary Figure S3C and Supplementary Table S4). In addition, the range of the maximum probability of factors of the three studied species is shown in Supplementary Table S4.

### 3.2. Current Distribution Regions of the Three Species

**Figure 3:**
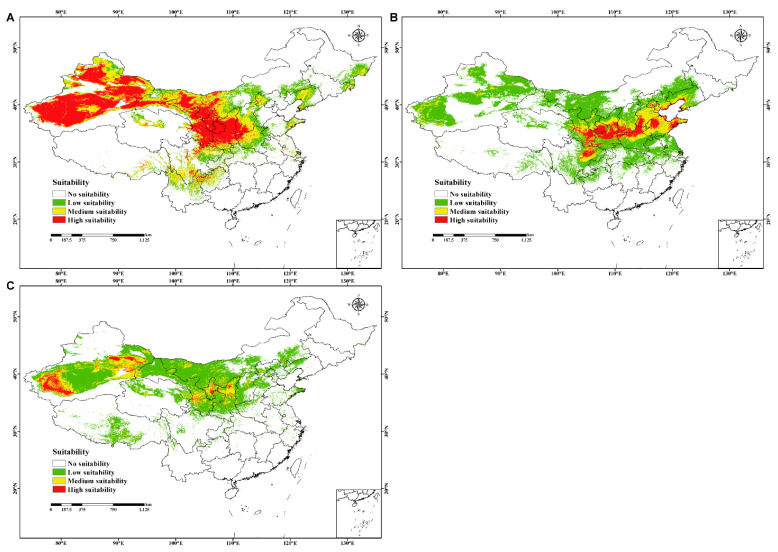
The current potential geographical distribution for three studied species in China. (**A**–**C**) represent *C. chrysosperma* (no suitability *p* ≤ 0.2812; low suitability 0.2812 < *p* ≤ 0.4; medium suitability 0.4 < *p* ≤ 0.6; high suitability *p* > 0.6, *p* = probability), *C. mali* (no suitability *p* ≤ 0.0887; low suitability 0.0887 < *p* ≤ 0.4; medium suitability 0.4 < *p* ≤ 0.6; high suitability *p* > 0.6, *p* = probability) and *C. nivea* (no suitability *p* ≤ 0.1184; low suitability 0.1184 < *p* ≤ 0.4; medium suitability 0.4 < *p* ≤ 0.6; high suitability *p* > 0.6, *p* = probability), respectively.

The prediction results of *C. chrysosperma*, *C. mali*, and *C. nivea* in China fit the known distribution in native ranges, reflecting the detection efficiency of our models. Among them, *C. chrysosperma* had the largest possible distribution area, covering approximately 36.2 × 10^5^ km^2^, accounting for 37.7% of the Chinese land surface area. The high- and medium-suitability areas primarily included Xinjiang, Gansu, Ningxia, Shaanxi, Sichuan, Midwest Nei Monggol, south-central Shanxi, and north Henan, with sporadic distributions in of Liaoning, Eastern Heilongjiang, Jilin, Shandong, Hebei, Qinghai, Xizang, Yunnan, and Guizhou (Figure 3A and Supplementary Table S5). Meanwhile, *C. mali* had a relatively narrower distribution range than *C. chrysosperma*. Specifically, *C. mali* covered approximately 32.4 × 10^5^ km^2^, accounting for 33.8% of the Chinese land surface area. The high- and medium-suitability areas primarily involved Gansu, Shaanxi, Shanxi, Hebei, Shandong, Sichuan, Henan, and Tianjin, with sporadic distributions in Ningxia, Liaoning, Beijing, and Xinjiang (Figure 3B and Supplementary Table S6). Moreover, *C. nivea* covers the smallest possible distribution area of approximately 27.9 × 10^5^ km^2^, accounting for 29.1%. The high- and medium-suitability areas were mainly in Xinjiang, Gansu, Ningxia, and Shaanxi, with sporadic distributions in Qinghai, Xizang, Shanxi, Hebei, and Nei Monggol (Figure 3C and Supplementary Table S7).

### 3.3. Future Distribution Changes of the Three Species

North Qinghai and most of Xinjiang, Gansu, Ningxia, Shaanxi, Nei Monggol, and Sichuan are mainly possible distribution zones for *C. chrysosperma* with climate change (Supplementary Figure S4). The medium-suitability habitat is predicted to expand as a result of climate change. At the same time, the low- and high-suitability habitats will generally decrease to varying degrees. The average suitable area (39.61%) would increase under the 12 future climatic scenarios compared to the current time (37.75%) (Supplemental Table S5). Although *C. mali* has a narrower range of acceptable habitats than *C. chrysosperma*, it may adapt to wetter and warmer climates, such as those in Henan, Shandong, Jiangsu, Hubei, Sichuan, and Guizhou (Supplementary Figure S5). In the 12 future climate scenarios, the high-, medium-, and low-suitability habitats are expected to increase remarkably, and the average suitable areas (44.29%) would increase compared with the current time (33.79%) (Supplementary Table S6). The range of potentially suitable habitats for *C. nivea* was the smallest, occurring only in Xinjiang, Gansu, Nei Monggol, Ningxia, Xizang, Shaanxi, and Qinghai (Supplementary Figure S6). The high-, medium-, and low-suitability habitats are predicted to expand due to climate change. The average suitable area (34.08%) would increase compared with the current period (29.13%) (Supplementary Table S7).

Supplementary Figures S7–S9 and Tables S8–S10 illustrate the contraction and expansion of the potential range of *C. chrysosperma*, *C. mali*, and *C. nivea* in China with climate change. Although the extent of the possible range changes varied among the SSPs, all the SSPs anticipated northward shifts in the prospective distributions of the three species. *C. chrysosperma* migrated into high-latitude and high-altitude habitats. At the same time, *C. mali* and *C. nivea* migrated to high latitudes and locations, but their tendency to migrate to high altitudes was not great. The expansion area under a high-emission scenario for the three studied species was larger than that under a low-emission scenario compared to the current climatic conditions. The contraction area under a low-emission scenario was lower than that under a high-emission scenario. The results indicate that future climate change will considerably alter the range of appropriate habitats for the three species.

### 3.4. Comparisons of Overlapping Areas, Distribution Centroids, and Ecological Niches for the Three Studied Species

**Figure 4:**
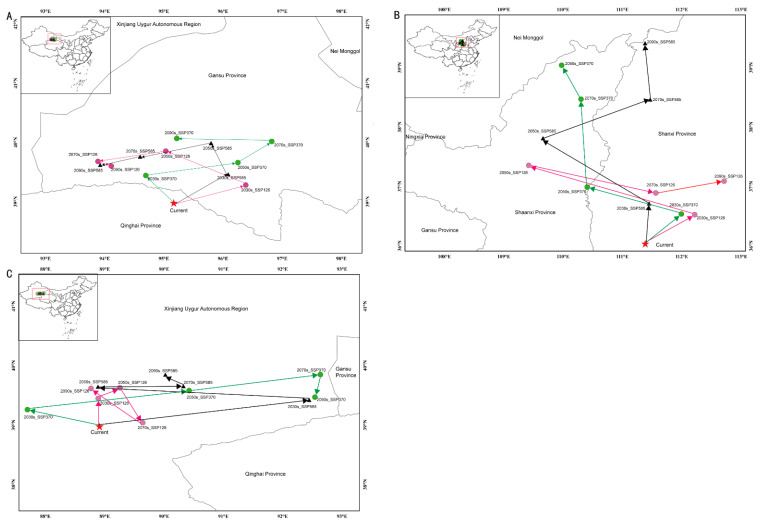
Centroid shifts of potential suitable area for three studied species under different climatic scenarios in China. Red star indicates the centroids of the suitable habitats of each species under current climate. Dots and triangles represent the centroids of the suitable habitats of each species under different future climate scenarios. (**A**–**C**) represent *C. chrysosperma*, *C. mali* and *C. nivea*, respectively.

The predictions of the Maxent model under the current climate conditions suggested that the appropriate areas for the three studied species overlapped to varied degrees with the suitable area (Figure 2). The overlapping area between *C. chrysosperma* and *C. nivea* was the greatest (28.6 × 10^4^ km^2^), followed by *C. mali* (24.9 × 10^4^ km^2^). The distribution centroids of *C. chrysosperma, C. mali*, and *C. nivea* were distributed in Qinghai (95.167233° N, 38.975466° E), Shanxi (111.386901° N, 36.032607° E), and Xinjiang (88.912738° N, 38.976171° E), respectively, under the current climate change scenario (Figure 4 and Table 2). Under various climate change scenarios, the centers of the appropriate habitat areas of the three species were expected to shift to high-latitude regions.

**Figure 5:**
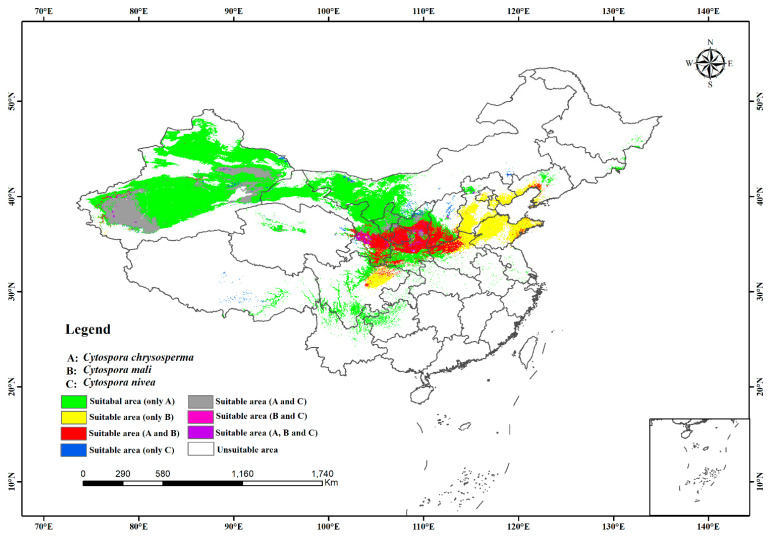
Niche and range overlap of the potential distribution habitats of the three species.

Based on the predicted suitable habitat, we estimated the niche overlap index and mapped the overlap of the potential distribution habitats of the three studied species in China. According to Table 3, there was less niche overlap between *C. chrysosperma* and *C. mali* (D = 0.6093, I = 0.8730) than between *C. chrysosperma* and *C. nivea* (D = 0.6848, I = 0.8956). However, The range overlap between *C. chrysosperma* and *C. nivea* was higher (0.9246) than that between *C. chrysosperma* and *C. mali* (0.5285). There was niche overlap and separation for *C. mali* and *C. nivea* (D = 0.5488, I = 0.8342) (Table 2). In China, the main niches of *C. chrysosperma* overlapped with the niches of *C. mali* and *C. nivea*. These three species coexist mainly in the northwest and north regions. The niche of *C. mali* could reach the warm and wet eastern region. In contrast, the niches of *C. chrysosperma* and *C. nivea* mainly existed in drought with low rainfall areas in western China (Figure 5).

## 4. Discussion

Due to the unique growth features of fungi in comparison to other taxa such as animals and plants, species distribution model (SDM) research on fungi began quite late and is scarce [67]. However, with the rapid expansion of disciplines like statistics, computer technology, and geographic information systems in recent years, fungi SDMs have notably increased in the study of pathogenic microfungi, lichens, and macrofungi [5]. Although there are many uses for fungi SDMs, the majority of them are grouped into three main categories: (1) investigating environmental factors that influence occurrence, (2) forecasting occurrence in specific regions, and (3) utilizing fungus as a model organism to investigate ecological or methodological ideas [5]. For example, Ajene et al. [44] employed three species distribution models (BIOCLIM, MaxEnt and Boosted Regression Trees) to predict the current and future potential distribution of *Candidatus Liberibacter asiaticus* in Africa, and the potential global distribution of *Candidatus Liberibacter africanus*, using long-term bioclimatic variables. Ejaz et al. [47] utilized four SDMs (GLM, generalized linear model; GAM, generalized additive model; GBM, generalized boosting model; and MaxEnt, maximal entropy) to investigate the suitability of *Fusarium* spp. disease around the world under climate change.

Currently, there is little information on the prevalence and occurrence of *Cytospora* canker in large-scale locations. During 2019–2023, our group conducted some work on the epidemic dynamics of *Cytospora* canker in Xinjiang Uygur Autonomous Region, China, focusing primarily on data gathering and field disease monitoring, laying the fundamental work for constructing prediction models. In this work, we used Maxent to investigate the possible habitat suitability of *C. chrysosperma*, *C. mali*, and *C. nivea* under present and future climatic circumstances. The AUC values all exceeded 0.9, indicating excellent accuracy. The climatic variables, particularly the temperature-related ones, are prominent factors for the studied species when selecting their habitats, such as the annual mean temperature (Bio 1), mean temperature of the coldest quarter (Bio 11), and mean temperature of the driest quarter (Bio 9), followed by the precipitation factors. The topographical factors (altitude, slope, and aspect) have weaker effects on species distribution. This could be as a result of the three investigated species being impacted by environmental factors disproportionately [68]. 

In general, the effects of terrain and soil factors on species distribution are frequently limited to smaller spatial scales and have a stronger impact on pathogen host plants, whereas climate factors, including temperature and precipitation, have the opposite effect [69]. Previous field investigations have indicated that the three examined species mostly infect host plants via various wounds, temperature is the most important environmental factor influencing the occurrence and prevalence of *Cytospora* canker disease, and precipitation aids spore dispersal but has a minor impact [70]. Our findings demonstrated that three of the examined species were highly adapted to the regions of north, northeast, and northwest China, implying that the dry and cold circumstances in China are always good for these taxa. The northern area of China, as is well known, has a temperate continental climate and a temperate monsoon climate, with low annual rainfall, cold and dry winters, and high summer temperatures, and wounds are prone to forming in this harsh climate environment [71]. Furthermore, the ideal temperature for the three examined species is 25–28 °C [24], and strains cannot develop when the ambient temperature is below 5 °C or above 35 °C [72]. Therefore, there are often two infection peaks for *Cytospora* canker each year: one from March to May and another from September to October [24]. Infections from November onward are extremely rare. This is in line with the finding that temperature affects the distribution of the three species under study more so than precipitation. Additionally, the models confirmed that *C. chrysosperma* was the most widely dispersed species compared to *C. mali* and *C. nivea*, implying the greatest potential for harm within the research area, consistent with the findings of previous resource surveys [13,15,19]. However, the predicted potential distributions of the three studied species were much more comprehensive than currently documented. Therefore, additional survey efforts are required to corroborate our findings.

Climate change has been documented as global warming and rising temperatures, which could lead to the redistribution or extinction of species in the future [73,74]. Many studies have shown that habitat migrations are primarily observed and expected to move toward higher latitudes and altitudes under a warming climate [6,32]. Our results showed a similar trend for *C. chrysosperma*. However, there is a trend for *C. mali* and *C. nivea* to shift toward higher latitudes but not toward higher altitudes, suggesting that the altitude either does not affect their distribution or has a minor impact. This may be because each species has unique physiological properties. Thus, adaptations to future climates will differ and depend on the ecological traits of the species. Additionally, the general trend for suitable regions in China is increasing in all of the warming scenarios, and habitat fragmentation is a crucial problem based on the range shift comparison of the present and future (Supplementary Figures S6–S8 and Tables S8–S10). Unsuitable regions may become suitable, whereas moderate and marginal areas may become ideal-suitability areas. This indicates that China must deal with a more serious *Cytospora* canker crisis.

Niche breadth and niche overlap are essential indicators for describing the actual ecological niche of a species, which, to some extent, reflects the characteristics of plants and their adaptability to the environment [75]. Niche breadth refers to the total utilization of various environmental resources by species. In contrast, niche overlap is the degree of similarity and competition between species in resource utilization [76]. In general, species with wide ecological niches may be more resilient to climate change and more able to adapt to it than species with narrow ecological niches [77]. *C. chysosperma* had the widest niche breadth among the three species, followed by *C. nivea*. The niche and range overlaps between *C. chysosperma* and *C. nivea* were high. Meanwhile, the geographical diffusion centers of *C. chysosperma* and *C. nivea* were also relatively close. However, due to the higher competitive ability of *C. chysosperma* than *C. nivea*, the potential diffusion range was significantly higher than that of *C. nivea.* Although the niche overlap and range overlap of *C. chysosperma* and *C. mali* were slightly lower than those of *C. chysosperma* and *C. nivea*, the centroids of *C. chysosperma* and *C. mali* were far away (Figure 4 and Table 2). With climate change, competition among these species may intensify as their harmful areas in China increase, and spatial overlap is expected to increase.

The three studied species have different potential diffusion zones in northeast, northwest, north, and southwest China. Therefore, it is necessary to develop corresponding prevention and control measures for the potential diffusion characteristics and key diffusion areas of each species to avoid unknown diffusion hazards. The border area between Gansu, Xinjiang, and Qinghai must be given special attention because the current and future core distributions of *C. chrysosperma* and *C. nivea* are located in this area. In the future, the monitoring of *C. nivea* should be strengthened in north Xinjiang, central and east Nei Monggol, central and west Xizang, and north Qinghai. The areas around Bohai Bay, north Loess Plateau, north Qinghai, and north Xinjiang should focus on strengthening the dynamic monitoring and quarantine of *C. mali*. The dynamic monitoring and quarantine of *C. chrysosperma* should be strengthened in the western loess plateau, eastern, northeast, and western Sichuan plateau, western Xizang, Tarim Basin, and Junggar Basin. 

There are indeed some limitations to our study. First, the accuracy of species occurrence data, especially from published sources, increases the forecasting uncertainty. Due to the lack of latitude and longitude coordinates, precise distribution locations were determined by searching for place names with coordinate positioning software, which may have resulted in geographical mistakes. Second, the model was based on an ideal niche and did not consider the effects of additional elements, such as the species self-diffusion ability, sampling biases, uncertainties in identification and taxonomy, host conditions, species interactions, human activities, variety type, medication frequency, and socioeconomic structure. Third, prediction precision still has some limitations, although the individual SDM model in this study demonstrated great prediction accuracy. The accuracy and performance levels of SDMs vary considerably between approaches and species. Some research has shown that approaches that integrate numerous individual models produce robust estimates of the possible distributions of species, which can be used to increase the model prediction accuracy. The causes mentioned above may result in discrepancies between the expected and actual distributions. Therefore, future research must address these issues that impact the precision of model predictions.

## 5. Conclusions

Based on the worldwide distribution records and bioclimatic data in this study, we used the maximum entropy model (Maxent) to predict the potentially suitable areas of *C. chrysosperma*, *C. mali*, and *C. nivea* in China. The findings revealed that temperature-related climate factors limit the three species’ current distribution ranges. The three studied species were highly suitable for northeast, northwest, north, and southwest China. Under future climate scenarios, the distribution areas of the three species are expected to increase, and the centers of the appropriate habitat areas of the three species will shift to high-latitude regions. The three species coexist in China, primarily in the northwest and north regions. The ecological niches of *C. chrysosperma, C. nivea*, and *C. mali* are more similar. The distribution range of *C. mali* can reach the warmer and wetter eastern region, whereas *C. chrysosperma* and *C. nivea* are primarily found in drought-prone areas with little rainfall. Our findings can help farmers and planners develop methods to avoid the spread of *Cytospora* spp. and calculate the costs of applying pesticides to reduce contamination and boost yields.

## Figures and Tables

**Table 1:** Results of MaxEnt models with optimized parameters developed for three studied species.

	*C. chrysosperma*	*C. mali*	*C. nivea*
n	374	164	166
RM	0.4	1.5	1.2
FC	LQP	LQH	QT
^n^train	280	123	124
^n^test	94	41	42
Train. AUC	0.9186	0.9892	0.9654
avg.test.AUC	0.921	0.987	0.946
AICc	10,614.12	3921.17	4564
delta.AICc	0	0	0
TSS	0.797	0.898	0.802

Note: n: number of species occurrences used in modelling; RM: regularization multiplier; FC: feature class; ^n^train: number of species occurrences used in training the model; ^n^test: number of species occurrences used in testing the model; Train. AUC: area under the curve for training data; avg.test.AUC: area under the curve for testing data. The AUC values are the means across 10 replicates.

**Table 2:** Centroid migration of suitable areas under future climatic conditions for target species.

Climate Scenario	Period	*C. chrysosperma*				*C. mali*				*C. nivea*			
		Centroid Coordinates		Direction	Migration Distance (between Two Adjacent Decades)/km	Centroid Coordinates		Direction	Migration Distance (between Two Adjacent Decades)/km	Centroid Coordinates		Direction	Migration Distance (between Two Adjacent Decades)/km
		Longitude/°E	Latitude/°N			Longitude/°E	Latitude/°N			Longitude/°E	Latitude/°N		
Current	1970–2000	95.167233	38.975466			111.386901	36.032607			88.912738	38.976171		
SSP126	2021–2040/2030s	96.384066	39.277208	Northeast	121.2	112.219521	36.523203	Northeast	94.9	88.8893	39.451271	North	47.3
	2041–2060/2050s	95.031757	39.852458	North	87.9	109.422709	37.353043	Northwest	233.1	89.254026	39.630005	Northeast	72.8
	2061–2080/2070s	93.884674	39.677991	Northwest	142	111.562266	36.889047	North	89.9	89.63857	39.039926	East	70.3
	2081–2100/2090s	94.108974	39.599974	Northwest	119.5	112.715643	37.086877	Northeast	167.9	88.766862	39.619537	North	65.4
SSP370	2021–2040/2030s	94.694191	39.439458	Northwest	64.9	111.993312	36.533684	Northeast	78.1	87.694677	39.260652	Northwest	120.9
	2041–2060/2050s	96.251491	39.65637	Northeast	124.5	110.404936	36.987467	Northwest	136.6	90.421173	39.58158	Northeast	157.5
	2061–2080/2070s	96.823543	40.015276	Northeast	190.1	110.302057	38.467943	Northwest	269.6	92.634879	39.851333	Northeast	369.5
	2081–2100/2090s	95.217266	40.065931	North	108	109.974273	39.039433	Northwest	334.7	92.541241	39.471202	Northeast	353.5
SSP585	2021–2040/2030s	96.086851	39.460776	Northeast	101	111.449265	36.712647	North	70.4	92.446198	39.424921	Northeast	343.8
	2041–2060/2050s	95.797745	39.988979	Northeast	117.3	109.659796	37.813257	Northwest	247.1	88.884588	39.648996	North	66.8
	2061–2080/2070s	94.599344	39.759687	Northwest	95.1	111.468502	38.464054	North	248.2	90.324758	39.656755	Northeast	152.1
	2081–2100/2090s	93.923118	39.618821	Northwest	136	111.381883	39.413539	North	342.8	90.021173	39.851333	Northeast	137.7

**Table 3:** Niche and range overlap of the potential distribution habitats of the three species.

Species	Niche Overlap (D)			Niche Overlap (I)			Range Overlap			Niche Breadth
	*C. chrysosperma*	*C. mali*	*C. nivea*	*C. chrysosperma*	*C. mali*	*C. nivea*	*C. chrysosperma*	*C. mali*	*C. nivea*	
*C. chrysosperma*	1	0.6093	0.6848	1	0.8730	0.8956	1	0.5285	0.9246	0.49
*C. mali*	-	1	0.5488	-	1	0.8342	-	1	0.0912	0.31
*C. nivea*	-	-	1	-	-	1	-	-	1	0.34

Note: D = Schoener’s D, I = Hellinger’s-based I.

## Data Availability

The original contributions presented in the study are included in the article/Supplementary Material. Further inquiries can be directed to the corresponding author.

## Supplementary Materials

The following supporting information can be downloaded at https://www.mdpi.com/article/10.3390/jof10010038/s1: Figure S1 Correlation analysis of various environmental factors. Figure S2 Jackknife test of variable importance Regularized training gain. Figure S3 Response curves for predictors in MaxEne model. Figure S4 The future potential geographical distributions of *C. chrysosperma* in China under future climatic conditions. Figure S5 The future potential geographical distributions of *C. mali* in China under future climatic conditions. Figure S6 The future potential geographical distributions of *C. nivea* in China under future climatic conditions. Figure S7 Adaptability changes in *C. chrysosperma* under different future climate scenarios. Figure S8 Adaptability changes in *C. mali* under different future climate scenarios. Figure S9 Adaptability changes in *C. nivea* under different future climate scenarios. Table S1 Geographical distributions of *C. chrysosperma*, *C. mali* and *C. nivea* species sampled in this study. Table S2 Environmental variables for current period model analysis. Table S3 List of selected environmental variables for each species. Table S4 Key climatic factors influencing habitat distributions of three species. Table S5 Dynamics of changes in distribution area of *C. chrysosperma* under different climate scenarios. Table S6 Dynamics of changes in distribution area of *C. mali* under different climate scenarios. Table S7 Dynamics of changes in distribution area of *C. nivea* under different climate scenarios. Table S8 Percentage of distribution changes of *C. chrysosperma* between current and future climate-change scenarios. Table S9 Percentage of distribution changes of *C. mali* between current and future climate-change scenarios. Table S10 Percentage of distribution changes of *C. nivea* between current and future climate-change scenarios.
