# Supplementary material for: Comparative Assessment of Habitat Suitability and Niche Overlap of Three Cytospora Species in China
Source: J Fungi (Basel). 2024 Jan 3;10(1):38. doi: 10.3390/jof10010038 (PMC10817479; doi:10.3390/jof10010038)
Supplement: Supplementary file 1 [file jof-10-00038-s001.zip › Table S1.pdf]

Table S1. Geographical distributions of *C. chrysosperma*, *C. mali* and *C. nivea* species sampled in this study

Species	Longitude (°)	Latitude (°)	Species	Longitude (°)	Latitude (°)
C.chrysosperma	-0.520833	50.854167	*C.chrysosperma*	112.729167	35.104167
C.chrysosperma	-0.895833	39.604167	*C.chrysosperma*	113.354167	40.062500
C.chrysosperma	-1.187500	53.395833	*C.chrysosperma*	114.395833	38.104167
C.chrysosperma	-1.187500	53.437500	*C.chrysosperma*	114.937500	34.479167
C.chrysosperma	-1.479167	52.270833	*C.chrysosperma*	115.104167	34.729167
C.chrysosperma	-100.270833	51.187500	*C.chrysosperma*	116.354167	37.437500
C.chrysosperma	-100.937500	37.062500	*C.chrysosperma*	116.937500	30.479167
C.chrysosperma	-101.729167	23.854167	*C.chrysosperma*	117.187500	31.895833
C.chrysosperma	-103.895833	42.687500	*C.chrysosperma*	117.229167	36.187500
C.chrysosperma	-104.229167	34.437500	*C.chrysosperma*	117.437500	36.770833
C.chrysosperma	-104.812500	38.854167	*C.chrysosperma*	118.187500	30.062500
C.chrysosperma	-104.854167	44.187500	*C.chrysosperma*	118.354167	31.229167
C.chrysosperma	-104.937500	38.854167	*C.chrysosperma*	118.812500	32.062500
C.chrysosperma	-105.104167	40.604167	*C.chrysosperma*	12.020833	54.979167
C.chrysosperma	-105.229167	39.729167	*C.chrysosperma*	12.062500	54.979167
C.chrysosperma	-105.229167	39.770833	*C.chrysosperma*	12.395833	55.687500
C.chrysosperma	-105.562500	28.312500	*C.chrysosperma*	12.562500	52.437500
C.chrysosperma	-105.604167	41.312500	*C.chrysosperma*	127.729167	43.645833
C.chrysosperma	-108.187500	45.979167	*C.chrysosperma*	127.812500	43.687500
C.chrysosperma	-108.520833	45.770833	*C.chrysosperma*	13.312500	47.312500
C.chrysosperma	-108.895833	45.395833	*C.chrysosperma*	13.354167	65.104167
C.chrysosperma	-109.020833	45.229167	*C.chrysosperma*	13.437500	52.312500
C.chrysosperma	-110.354167	46.895833	*C.chrysosperma*	13.895833	65.812500
C.chrysosperma	-111.604167	57.187500	*C.chrysosperma*	138.895833	-35.270833
C.chrysosperma	-111.812500	41.770833	*C.chrysosperma*	14.437500	46.562500
C.chrysosperma	-112.020833	46.604167	*C.chrysosperma*	140.979167	43.020833
C.chrysosperma	-112.187500	38.395833	*C.chrysosperma*	141.062500	39.645833
C.chrysosperma	-112.229167	38.437500	*C.chrysosperma*	141.354167	43.020833
C.chrysosperma	-112.270833	38.437500	*C.chrysosperma*	141.354167	43.062500
C.chrysosperma	-112.687500	33.937500	*C.chrysosperma*	15.354167	66.562500
C.chrysosperma	-112.729167	46.395833	*C.chrysosperma*	15.854167	65.104167
C.chrysosperma	-116.645833	46.937500	*C.chrysosperma*	169.187500	-45.062500
C.chrysosperma	-116.937500	46.854167	*C.chrysosperma*	169.312500	-45.229167
C.chrysosperma	-117.187500	46.729167	*C.chrysosperma*	169.395833	-45.270833
C.chrysosperma	-117.354167	46.645833	*C.chrysosperma*	17.104167	68.479167
C.chrysosperma	-118.145833	47.645833	*C.chrysosperma*	170.479167	-44.020833
C.chrysosperma	-118.229167	46.604167	*C.chrysosperma*	172.687500	-43.395833
C.chrysosperma	-118.854167	46.645833	*C.chrysosperma*	175.270833	-40.604167
C.chrysosperma	-119.312500	34.354167	*C.chrysosperma*	175.604167	-40.354167
C.chrysosperma	-119.520833	36.604167	*C.chrysosperma*	178.020833	-38.645833

C.chrysosperma	-119.645833	36.770833	*C.chrysosperma*	18.354167	68.895833
C.chrysosperma	-121.270833	38.562500	*C.chrysosperma*	18.729167	69.020833
C.chrysosperma	-121.479167	45.729167	*C.chrysosperma*	19.645833	68.895833
C.chrysosperma	-121.812500	37.270833	*C.chrysosperma*	20.104167	69.104167
C.chrysosperma	-121.895833	37.729167	*C.chrysosperma*	21.104167	52.354167
C.chrysosperma	-121.937500	37.437500	*C.chrysosperma*	21.270833	69.562500
C.chrysosperma	-121.937500	37.645833	*C.chrysosperma*	22.895833	70.020833
C.chrysosperma	-121.937500	37.937500	*C.chrysosperma*	23.979167	61.562500
C.chrysosperma	-122.062500	37.937500	*C.chrysosperma*	24.937500	60.187500
C.chrysosperma	-122.187500	47.104167	*C.chrysosperma*	25.145833	61.062500
C.chrysosperma	-122.270833	37.854167	*C.chrysosperma*	25.687500	68.979167
C.chrysosperma	-122.270833	37.895833	*C.chrysosperma*	26.062500	-32.687500
C.chrysosperma	-122.479167	48.770833	*C.chrysosperma*	27.104167	62.062500
C.chrysosperma	-122.812500	45.729167	*C.chrysosperma*	27.937500	-26.687500
C.chrysosperma	-17.979167	65.895833	*C.chrysosperma*	28.312500	-26.187500
C.chrysosperma	-2.229167	37.229167	*C.chrysosperma*	29.687500	-30.062500
C.chrysosperma	-2.604167	36.812500	*C.chrysosperma*	30.270833	-29.562500
C.chrysosperma	-2.604167	42.354167	*C.chrysosperma*	41.437500	61.104167
C.chrysosperma	-2.812500	42.937500	*C.chrysosperma*	44.520833	40.145833
C.chrysosperma	-2.812500	52.104167	*C.chrysosperma*	6.104167	49.729167
C.chrysosperma	-3.562500	38.312500	*C.chrysosperma*	6.229167	46.229167
C.chrysosperma	-4.020833	56.229167	*C.chrysosperma*	6.895833	49.187500
C.chrysosperma	-4.520833	42.020833	*C.chrysosperma*	7.187500	47.229167
C.chrysosperma	-4.729167	42.520833	*C.chrysosperma*	7.479167	46.020833
C.chrysosperma	-5.854167	56.812500	*C.chrysosperma*	7.645833	51.354167
C.chrysosperma	-6.104167	36.687500	*C.chrysosperma*	7.812500	46.312500
C.chrysosperma	-6.520833	40.604167	*C.chrysosperma*	73.354167	61.270833
C.chrysosperma	-7.479167	41.562500	*C.chrysosperma*	73.437500	61.229167
C.chrysosperma	-71.104167	42.395833	*C.chrysosperma*	75.104167	39.145833
C.chrysosperma	-71.395833	46.854167	*C.chrysosperma*	76.020833	39.270833
C.chrysosperma	-72.937500	41.312500	*C.chrysosperma*	76.062500	39.270833
C.chrysosperma	-73.479167	45.520833	*C.chrysosperma*	76.104167	39.437500
C.chrysosperma	-75.020833	39.562500	*C.chrysosperma*	76.145833	37.020833
C.chrysosperma	-75.645833	45.395833	*C.chrysosperma*	76.145833	39.312500
C.chrysosperma	-75.687500	45.437500	*C.chrysosperma*	76.145833	39.770833
C.chrysosperma	-79.395833	43.687500	*C.chrysosperma*	76.187500	38.854167
C.chrysosperma	-79.645833	43.854167	*C.chrysosperma*	76.270833	39.229167
C.chrysosperma	-79.729167	44.895833	*C.chrysosperma*	76.520833	39.437500
C.chrysosperma	-80.104167	43.354167	*C.chrysosperma*	76.604167	37.770833
C.chrysosperma	-80.520833	43.187500	*C.chrysosperma*	76.604167	38.604167
C.chrysosperma	-80.895833	35.145833	*C.chrysosperma*	76.895833	37.062500
C.chrysosperma	-83.729167	42.229167	*C.chrysosperma*	76.895833	38.020833
C.chrysosperma	-86.020833	49.979167	*C.chrysosperma*	76.895833	39.145833
C.chrysosperma	-86.979167	46.354167	*C.chrysosperma*	76.979167	36.854167

C.chrysosperma	-87.812500	41.812500	*C.chrysosperma*	76.979167	38.145833
C.chrysosperma	-87.854167	41.812500	*C.chrysosperma*	77.020833	38.395833
C.chrysosperma	-89.395833	43.062500	*C.chrysosperma*	77.062500	38.229167
C.chrysosperma	-89.520833	45.687500	*C.chrysosperma*	77.104167	38.229167
C.chrysosperma	-89.645833	43.437500	*C.chrysosperma*	77.104167	38.395833
C.chrysosperma	-89.979167	48.229167	*C.chrysosperma*	77.104167	38.479167
C.chrysosperma	-90.354167	47.770833	*C.chrysosperma*	77.104167	38.645833
C.chrysosperma	-91.770833	43.312500	*C.chrysosperma*	77.145833	38.270833
C.chrysosperma	-93.104167	44.937500	*C.chrysosperma*	77.145833	38.479167
C.chrysosperma	-93.395833	45.229167	*C.chrysosperma*	77.187500	38.020833
C.chrysosperma	-93.604167	42.020833	*C.chrysosperma*	77.229167	38.187500
C.chrysosperma	-94.479167	43.645833	*C.chrysosperma*	77.270833	37.854167
C.chrysosperma	-95.562500	38.770833	*C.chrysosperma*	77.270833	38.104167
C.chrysosperma	-96.562500	39.187500	*C.chrysosperma*	77.270833	38.270833
C.chrysosperma	-96.687500	40.812500	*C.chrysosperma*	77.270833	38.312500
C.chrysosperma	-96.729167	39.312500	*C.chrysosperma*	77.312500	38.062500
C.chrysosperma	-96.812500	44.312500	*C.chrysosperma*	77.312500	38.187500
C.chrysosperma	-97.229167	41.687500	*C.chrysosperma*	77.312500	38.229167
C.chrysosperma	-97.354167	38.937500	*C.chrysosperma*	77.312500	39.687500
C.chrysosperma	-97.687500	38.562500	*C.chrysosperma*	77.354167	38.062500
C.chrysosperma	-98.562500	45.145833	*C.chrysosperma*	77.395833	37.729167
C.chrysosperma	-98.770833	38.020833	*C.chrysosperma*	77.395833	37.937500
C.chrysosperma	-99.270833	39.437500	*C.chrysosperma*	77.395833	38.770833
C.chrysosperma	-99.312500	39.354167	*C.chrysosperma*	77.437500	38.604167
C.chrysosperma	-99.312500	39.562500	*C.chrysosperma*	77.437500	38.645833
C.chrysosperma	-99.937500	41.312500	*C.chrysosperma*	77.479167	37.854167
C.chrysosperma	0.937500	41.645833	*C.chrysosperma*	77.479167	38.937500
C.chrysosperma	1.479167	42.520833	*C.chrysosperma*	77.520833	38.020833
C.chrysosperma	1.479167	52.604167	*C.chrysosperma*	77.520833	38.145833
C.chrysosperma	1.520833	42.520833	*C.chrysosperma*	77.520833	38.479167
C.chrysosperma	1.645833	50.645833	*C.chrysosperma*	77.520833	38.895833
C.chrysosperma	101.812500	33.479167	*C.chrysosperma*	77.645833	37.979167
C.chrysosperma	101.937500	36.770833	*C.chrysosperma*	77.687500	38.770833
C.chrysosperma	102.145833	36.479167	*C.chrysosperma*	77.687500	38.895833
C.chrysosperma	102.520833	36.437500	*C.chrysosperma*	77.854167	38.979167
C.chrysosperma	102.729167	36.604167	*C.chrysosperma*	77.979167	39.104167
C.chrysosperma	103.145833	34.604167	*C.chrysosperma*	78.270833	37.604167
C.chrysosperma	103.187500	34.062500	*C.chrysosperma*	78.270833	39.520833
C.chrysosperma	103.354167	36.604167	*C.chrysosperma*	78.354167	39.604167
C.chrysosperma	103.479167	34.604167	*C.chrysosperma*	78.395833	37.687500
C.chrysosperma	103.604167	34.312500	*C.chrysosperma*	78.562500	37.395833
C.chrysosperma	103.645833	34.979167	*C.chrysosperma*	78.812500	39.812500
C.chrysosperma	103.687500	34.937500	*C.chrysosperma*	78.895833	39.645833
C.chrysosperma	103.729167	35.229167	*C.chrysosperma*	78.937500	39.729167

C.chrysosperma	104.145833	34.687500	*C.chrysosperma*	79.104167	37.270833
C.chrysosperma	105.020833	37.479167	*C.chrysosperma*	79.104167	39.895833
C.chrysosperma	105.312500	37.520833	*C.chrysosperma*	79.270833	41.187500
C.chrysosperma	105.687500	34.479167	*C.chrysosperma*	79.354167	40.687500
C.chrysosperma	105.895833	37.854167	*C.chrysosperma*	79.395833	40.062500
C.chrysosperma	105.895833	38.104167	*C.chrysosperma*	79.604167	37.145833
C.chrysosperma	106.187500	36.187500	*C.chrysosperma*	79.645833	36.937500
C.chrysosperma	106.187500	38.229167	*C.chrysosperma*	79.645833	37.479167
C.chrysosperma	106.229167	36.020833	*C.chrysosperma*	79.645833	41.187500
C.chrysosperma	106.312500	38.854167	*C.chrysosperma*	79.687500	37.562500
C.chrysosperma	106.354167	39.062500	*C.chrysosperma*	79.729167	37.145833
C.chrysosperma	107.687500	35.729167	*C.chrysosperma*	79.979167	37.104167
C.chrysosperma	107.770833	34.270833	*C.chrysosperma*	8.020833	50.020833
C.chrysosperma	107.979167	35.770833	*C.chrysosperma*	8.145833	47.020833
C.chrysosperma	108.062500	34.270833	*C.chrysosperma*	8.270833	46.479167
C.chrysosperma	109.770833	34.562500	*C.chrysosperma*	8.395833	56.479167
C.chrysosperma	111.645833	40.729167	*C.chrysosperma*	8.937500	46.020833
C.chrysosperma	111.729167	40.812500	*C.chrysosperma*	80.020833	41.395833
C.chrysosperma	111.729167	40.854167	*C.chrysosperma*	80.062500	40.354167
C.chrysosperma	112.395833	32.937500	*C.chrysosperma*	80.104167	40.395833
C.chrysosperma	112.520833	35.104167	*C.chrysosperma*	80.145833	40.395833
C.chrysosperma	80.229167	41.354167	*C.chrysosperma*	80.187500	37.104167
C.chrysosperma	80.270833	40.937500	*C.chrysosperma*	80.187500	40.395833
C.chrysosperma	80.270833	41.062500	*C.chrysosperma*	80.187500	41.312500
C.chrysosperma	80.312500	40.812500	*C.chrysosperma*	80.229167	41.312500
C.chrysosperma	80.354167	37.645833	*C.chrysosperma*	89.979167	36.812500
C.chrysosperma	80.354167	40.645833	*C.chrysosperma*	9.312500	47.020833
C.chrysosperma	80.354167	41.104167	*C.chrysosperma*	9.604167	62.312500
C.chrysosperma	80.354167	41.270833	*C.chrysosperma*	92.854167	43.062500
C.chrysosperma	80.479167	40.645833	*C.chrysosperma*	92.895833	43.062500
C.chrysosperma	80.562500	40.770833	*C.chrysosperma*	92.937500	43.062500
C.chrysosperma	80.562500	41.312500	*C.chrysosperma*	93.229167	42.895833
C.chrysosperma	80.604167	41.354167	*C.chrysosperma*	93.437500	42.895833
C.chrysosperma	80.645833	40.604167	*C.chrysosperma*	93.479167	42.854167
C.chrysosperma	80.770833	36.187500	*C.chrysosperma*	93.520833	42.812500
C.chrysosperma	80.770833	40.520833	*C.chrysosperma*	93.604167	42.729167
C.chrysosperma	80.812500	36.979167	*C.chrysosperma*	93.729167	42.770833
C.chrysosperma	80.812500	41.354167	*C.chrysosperma*	93.770833	42.812500
C.chrysosperma	80.854167	40.437500	*C.chrysosperma*	93.895833	42.895833
C.chrysosperma	80.854167	41.354167	*C.chrysosperma*	93.937500	42.645833
C.chrysosperma	80.937500	40.437500	*C.chrysosperma*	93.937500	42.687500
C.chrysosperma	80.937500	40.520833	*C.chrysosperma*	93.979167	42.687500
C.chrysosperma	81.062500	36.979167	*C.chrysosperma*	84.895833	45.562500
C.chrysosperma	81.270833	40.562500	*C.chrysosperma*	85.604167	44.312500

C.chrysosperma	81.354167	36.937500	*C.chrysosperma*	86.020833	41.812500
C.chrysosperma	81.520833	36.854167	*C.chrysosperma*	86.104167	40.854167
C.chrysosperma	81.562500	36.854167	*C.chrysosperma*	86.479167	40.312500
C.chrysosperma	81.729167	41.729167	*C.chrysosperma*	86.520833	41.937500
C.chrysosperma	81.854167	36.312500	*C.chrysosperma*	87.145833	40.770833
C.chrysosperma	82.270833	41.729167	*C.chrysosperma*	87.479167	43.979167
C.chrysosperma	82.645833	43.770833	*C.chrysosperma*	87.520833	43.979167
C.chrysosperma	82.687500	37.020833	*C.chrysosperma*	87.562500	43.729167
C.chrysosperma	82.687500	37.062500	*C.chrysosperma*	87.562500	44.187500
C.chrysosperma	82.687500	41.187500	*C.chrysosperma*	88.645833	42.770833
C.chrysosperma	82.812500	41.104167	*C.chrysosperma*	88.645833	42.812500
C.chrysosperma	82.812500	41.604167	*C.chrysosperma*	88.937500	39.270833
C.chrysosperma	82.979167	46.770833	*C.chrysosperma*	83.354167	46.729167
C.chrysosperma	83.062500	41.937500	*C.chrysosperma*	84.687500	44.437500
C.chrysosperma	83.145833	41.312500	*C.chrysosperma*	84.729167	44.687500
C.nivea	-104.395833	44.645833	*C.nivea*	22.895833	62.020833
C.nivea	-104.729167	39.354167	*C.nivea*	23.687500	60.520833
C.nivea	-105.520833	38.104167	*C.nivea*	23.729167	61.437500
C.nivea	-106.145833	38.479167	*C.nivea*	23.770833	61.437500
C.nivea	-106.312500	38.479167	*C.nivea*	23.812500	61.479167
C.nivea	-111.270833	44.479167	*C.nivea*	24.062500	61.229167
C.nivea	-112.104167	46.229167	*C.nivea*	24.937500	60.187500
C.nivea	-122.187500	48.062500	*C.nivea*	25.062500	60.229167
C.nivea	-71.187500	42.395833	*C.nivea*	25.645833	68.895833
C.nivea	-72.687500	44.645833	*C.nivea*	25.687500	68.979167
C.nivea	-75.645833	39.729167	*C.nivea*	25.687500	69.229167
C.nivea	-76.312500	42.562500	*C.nivea*	25.979167	64.812500
C.nivea	-77.270833	42.854167	*C.nivea*	26.062500	61.937500
C.nivea	-77.937500	43.062500	*C.nivea*	26.145833	62.270833
C.nivea	-84.270833	39.270833	*C.nivea*	26.187500	64.770833
C.nivea	-84.395833	40.229167	*C.nivea*	27.104167	60.729167
C.nivea	-84.479167	42.729167	*C.nivea*	27.145833	65.520833
C.nivea	-86.979167	46.354167	*C.nivea*	29.854167	63.104167
C.nivea	-88.812500	47.979167	*C.nivea*	3.479167	50.520833
C.nivea	-92.354167	47.895833	*C.nivea*	31.270833	49.229167
C.nivea	-92.437500	46.645833	*C.nivea*	4.312500	52.104167
C.nivea	-92.979167	45.312500	*C.nivea*	4.354167	52.145833
C.nivea	-93.187500	44.979167	*C.nivea*	4.562500	52.354167
C.nivea	-93.395833	44.937500	*C.nivea*	4.645833	52.312500
C.nivea	-93.395833	45.229167	*C.nivea*	4.645833	52.604167
C.nivea	-95.229167	47.270833	*C.nivea*	4.645833	52.645833
C.nivea	-96.687500	40.812500	*C.nivea*	4.687500	52.645833
C.nivea	-98.354167	42.270833	*C.nivea*	4.729167	52.645833
C.nivea	-99.270833	39.437500	*C.nivea*	5.395833	51.979167

C.nivea	-99.312500	39.562500	*C.nivea*	6.062500	49.687500
C.nivea	1.604167	50.437500	*C.nivea*	6.062500	49.812500
C.nivea	10.062500	60.104167	*C.nivea*	6.062500	49.979167
C.nivea	10.104167	56.187500	*C.nivea*	6.145833	49.729167
C.nivea	10.145833	61.520833	*C.nivea*	6.187500	49.729167
C.nivea	10.187500	59.270833	*C.nivea*	6.437500	52.354167
C.nivea	10.187500	60.145833	*C.nivea*	6.645833	52.312500
C.nivea	10.270833	59.437500	*C.nivea*	7.937500	58.604167
C.nivea	10.312500	59.187500	*C.nivea*	7.979167	50.979167
C.nivea	10.312500	60.187500	*C.nivea*	7.979167	51.020833
C.nivea	10.354167	59.354167	*C.nivea*	76.187500	38.854167
C.nivea	10.395833	59.104167	*C.nivea*	76.270833	39.145833
C.nivea	10.395833	59.312500	*C.nivea*	76.604167	37.770833
C.nivea	10.395833	59.354167	*C.nivea*	76.895833	39.145833
C.nivea	10.395833	59.854167	*C.nivea*	77.270833	38.312500
C.nivea	10.437500	51.104167	*C.nivea*	77.312500	38.187500
C.nivea	10.437500	59.312500	*C.nivea*	77.979167	39.104167
C.nivea	10.479167	59.854167	*C.nivea*	78.395833	37.687500
C.nivea	10.645833	59.979167	*C.nivea*	79.104167	37.270833
C.nivea	10.770833	59.270833	*C.nivea*	79.604167	37.145833
C.nivea	10.770833	59.479167	*C.nivea*	79.687500	37.562500
C.nivea	10.770833	59.687500	*C.nivea*	79.729167	37.145833
C.nivea	10.937500	60.687500	*C.nivea*	8.020833	50.020833
C.nivea	103.145833	34.604167	*C.nivea*	8.020833	50.854167
C.nivea	103.729167	35.229167	*C.nivea*	8.104167	50.854167
C.nivea	106.187500	36.187500	*C.nivea*	8.145833	50.854167
C.nivea	106.187500	38.229167	*C.nivea*	8.270833	46.937500
C.nivea	109.687500	38.312500	*C.nivea*	8.270833	50.145833
C.nivea	11.020833	60.812500	*C.nivea*	8.479167	59.395833
C.nivea	11.187500	60.729167	*C.nivea*	8.687500	58.479167
C.nivea	11.645833	63.979167	*C.nivea*	80.187500	37.104167
C.nivea	11.729167	59.229167	*C.nivea*	80.645833	40.604167
C.nivea	11.937500	58.229167	*C.nivea*	80.770833	36.187500
C.nivea	13.562500	52.812500	*C.nivea*	81.145833	43.854167
C.nivea	13.604167	52.812500	*C.nivea*	81.354167	36.937500
C.nivea	13.854167	58.395833	*C.nivea*	81.562500	36.854167
C.nivea	14.062500	50.895833	*C.nivea*	82.812500	41.604167
C.nivea	14.062500	50.937500	*C.nivea*	82.979167	46.770833
C.nivea	14.145833	65.687500	*C.nivea*	83.312500	46.770833
C.nivea	14.437500	63.145833	*C.nivea*	86.020833	41.812500
C.nivea	14.812500	48.562500	*C.nivea*	87.145833	40.770833
C.nivea	14.979167	62.062500	*C.nivea*	89.979167	36.812500
C.nivea	15.270833	66.520833	*C.nivea*	9.020833	60.562500
C.nivea	15.645833	66.895833	*C.nivea*	9.020833	60.854167

C.nivea	16.312500	64.229167	*C.nivea*	9.562500	49.979167
C.nivea	17.145833	68.479167	*C.nivea*	9.562500	59.229167
C.nivea	17.604167	59.854167	*C.nivea*	9.687500	59.020833
C.nivea	18.187500	69.104167	*C.nivea*	93.812500	43.270833
C.nivea	18.312500	59.937500	*C.nivea*	20.270833	63.812500
C.nivea	19.687500	64.104167	*C.nivea*	21.104167	69.812500
C.nivea	19.979167	47.812500	*C.nivea*	21.312500	69.645833
C.nivea	20.020833	63.895833	*C.nivea*	21.479167	65.854167
C.nivea	20.187500	63.812500	*C.nivea*	22.020833	69.854167
C.nivea	20.229167	63.812500	*C.nivea*	22.770833	62.812500
C.mali	101.437500	36.062500	*C.mali*	117.020833	39.770833
C.mali	101.729167	36.604167	*C.mali*	117.229167	31.895833
C.mali	101.729167	36.645833	*C.mali*	118.479167	31.687500
C.mali	101.854167	36.562500	*C.mali*	118.687500	39.729167
C.mali	102.020833	35.937500	*C.mali*	119.854167	40.145833
C.mali	102.312500	36.479167	*C.mali*	119.937500	36.854167
C.mali	102.354167	35.895833	*C.mali*	119.937500	40.104167
C.mali	102.479167	35.854167	*C.mali*	120.312500	40.312500
C.mali	102.562500	36.437500	*C.mali*	120.395833	37.354167
C.mali	102.729167	36.645833	*C.mali*	120.437500	40.479167
C.mali	102.812500	36.354167	*C.mali*	120.479167	37.645833
C.mali	102.937500	36.270833	*C.mali*	120.562500	37.104167
C.mali	103.604167	27.354167	*C.mali*	120.687500	37.187500
C.mali	103.645833	27.354167	*C.mali*	120.729167	36.979167
C.mali	103.645833	27.437500	*C.mali*	120.729167	40.604167
C.mali	103.729167	33.937500	*C.mali*	120.770833	37.187500
C.mali	105.187500	34.604167	*C.mali*	120.812500	40.729167
C.mali	105.229167	34.854167	*C.mali*	120.854167	37.312500
C.mali	105.312500	34.187500	*C.mali*	120.937500	40.854167
C.mali	105.354167	34.770833	*C.mali*	120.979167	37.062500
C.mali	105.395833	34.229167	*C.mali*	121.312500	38.895833
C.mali	105.479167	34.270833	*C.mali*	121.812500	39.270833
C.mali	105.520833	34.562500	*C.mali*	121.979167	39.395833
C.mali	105.562500	34.562500	*C.mali*	122.062500	39.770833
C.mali	105.645833	34.604167	*C.mali*	122.104167	37.520833
C.mali	105.729167	34.479167	*C.mali*	122.145833	39.937500
C.mali	105.729167	35.104167	*C.mali*	132.020833	46.437500
C.mali	105.729167	35.229167	*C.mali*	140.979167	43.020833
C.mali	105.729167	35.270833	*C.mali*	141.062500	39.645833
C.mali	105.729167	35.479167	*C.mali*	141.354167	43.062500
C.mali	105.770833	34.520833	*C.mali*	141.687500	43.145833
C.mali	105.770833	35.187500	*C.mali*	75.854167	39.354167
C.mali	105.770833	35.312500	*C.mali*	77.145833	38.354167
C.mali	105.812500	34.687500	*C.mali*	79.937500	37.104167

C.mali	105.854167	34.562500	*C.mali*	80.270833	41.145833
C.mali	105.854167	35.062500	*C.mali*	80.270833	41.229167
C.mali	105.854167	37.645833	*C.mali*	80.312500	41.312500
C.mali	105.895833	34.479167	*C.mali*	81.854167	43.729167
C.mali	105.895833	34.520833	*C.mali*	81.895833	41.812500
C.mali	105.895833	34.645833	*C.mali*	82.979167	46.770833
C.mali	105.895833	35.104167	*C.mali*	87.604167	43.812500
C.mali	105.895833	37.854167	*C.mali*	88.145833	47.854167
C.mali	105.979167	34.437500	*C.mali*	89.229167	42.979167
C.mali	105.979167	34.645833	*C.mali*	93.520833	42.812500
C.mali	106.062500	38.479167	*C.mali*	109.312500	34.479167
C.mali	106.104167	35.187500	*C.mali*	109.437500	35.770833
C.mali	106.312500	40.187500	*C.mali*	109.562500	35.854167
C.mali	106.437500	34.937500	*C.mali*	109.604167	35.604167
C.mali	106.895833	35.479167	*C.mali*	109.604167	35.687500
C.mali	106.937500	35.562500	*C.mali*	109.645833	35.520833
C.mali	107.062500	35.395833	*C.mali*	110.187500	36.062500
C.mali	107.229167	35.479167	*C.mali*	110.604167	35.229167
C.mali	107.270833	36.687500	*C.mali*	110.645833	35.354167
C.mali	107.354167	35.604167	*C.mali*	110.687500	34.645833
C.mali	107.479167	35.562500	*C.mali*	110.729167	35.229167
C.mali	107.604167	35.312500	*C.mali*	110.854167	34.520833
C.mali	107.604167	35.687500	*C.mali*	110.895833	34.520833
C.mali	107.687500	35.729167	*C.mali*	110.937500	34.437500
C.mali	107.770833	35.729167	*C.mali*	110.937500	34.520833
C.mali	107.770833	35.895833	*C.mali*	110.937500	34.770833
C.mali	107.812500	35.187500	*C.mali*	111.062500	34.062500
C.mali	107.812500	35.770833	*C.mali*	111.104167	34.395833
C.mali	107.812500	35.812500	*C.mali*	111.104167	34.729167
C.mali	107.854167	35.395833	*C.mali*	111.187500	34.770833
C.mali	107.854167	35.645833	*C.mali*	111.187500	34.979167
C.mali	107.895833	35.604167	*C.mali*	111.229167	34.312500
C.mali	107.979167	35.645833	*C.mali*	111.229167	34.895833
C.mali	108.062500	34.270833	*C.mali*	111.645833	34.395833
C.mali	108.145833	34.687500	*C.mali*	112.312500	32.937500
C.mali	108.187500	34.104167	*C.mali*	112.520833	37.895833
C.mali	108.187500	34.354167	*C.mali*	112.729167	34.937500
C.mali	108.229167	35.770833	*C.mali*	114.562500	34.479167
C.mali	108.354167	34.520833	*C.mali*	114.562500	37.062500
C.mali	108.437500	34.645833	*C.mali*	114.562500	38.437500
C.mali	108.604167	34.437500	*C.mali*	114.770833	35.645833
C.mali	109.062500	35.604167	*C.mali*	115.104167	31.770833
C.mali	109.270833	35.562500	*C.mali*	115.104167	40.604167
C.mali	115.520833	40.437500	*C.mali*	115.145833	38.854167

C.mali	115.812500	38.437500	*C.mali*	115.187500	34.645833
C.mali	116.270833	37.687500	*C.mali*	115.229167	37.937500
C.mali	116.770833	40.020833	*C.mali*	115.229167	40.354167
C.mali	116.937500	30.479167	*C.mali*	115.520833	38.770833
