# Supplementary material for: Comparative Assessment of Habitat Suitability and Niche Overlap of Three Cytospora Species in China
Source: J Fungi (Basel). 2024 Jan 3;10(1):38. doi: 10.3390/jof10010038 (PMC10817479; doi:10.3390/jof10010038)
Supplement: Supplementary file 1 [file jof-10-00038-s001.zip › Table S10.pdf]

Table S10. Percentage of distrbution changes of *C. nivea* between current and future climate-change scenarios

Scenario	Period	Extended area (×10^4/km^2)	Shrink area (×10^4/km^2)	Stable area (×10^4/km^2)
Current				
SSP126	2030S	9.758	4.341	27.878
	2050S	24.085	3.37	28.848
	2070S	13.376	2.896	29.322
	2090S	13.67	3.811	28.407
SSP370	2030S	5.144	9.902	22.316
	2050S	27.599	2.661	29.558
	2070S	31.866	4.198	28.019
	2090S	45.409	2.599	29.619
SSP585	2030S	19.197	3.193	29.026
	2050S	19.149	4.115	28.103
	2070S	27.623	4.596	27.622
	2090S	32.555	5.078	27.14
