# Supplementary material for: Comparative Assessment of Habitat Suitability and Niche Overlap of Three Cytospora Species in China
Source: J Fungi (Basel). 2024 Jan 3;10(1):38. doi: 10.3390/jof10010038 (PMC10817479; doi:10.3390/jof10010038)
Supplement: Supplementary file 1 [file jof-10-00038-s001.zip › Table S2.pdf]

Table S2. Environmental variables for current period model analysis

Type	Code	Description	Unit
	bio1	Annual mean temperature	℃
	bio2	Mean diurnal range (Mean of monthly (max.temp.-min.temp.)	℃
	bio3	Isothermality (bio2 / bio7) (× 100)	
	bio4	Temperature seasonality (standard deviation*100)	
	bio5	Max temperature of the warmest month	℃
	bio6	Min temperature of the coldest month	℃
	bio7	Temperature annual range (bio5- bio6)	℃
	bio8	Mean temperature of the wettest quarter	℃
	bio9	Mean temperature of the driest quarter	℃
Climate	bio10	Mean temperature of the warmest quarter	℃
	bio11	Mean temperature of the coldest quarter	℃
	bio12	Annual precipitation	mm
	bio13	Precipitation of the wettest month	mm
	bio14	Precipitation of the driest month	mm
	bio15	Precipitation seasonality (Coefficient of variation)	
	bio16	Precipitation of the wettest quarter	mm
	bio17	Precipitation of the driest quarter	mm
	bio18	Precipitation of the warmest quarter	mm
	bio19	Precipitation of coldest quarter	mm
	t-ph	Topsoil pH (H_2O)	
	t-clay	Topsoil clay fraction	%
	t-teb	Topsoil TEB	cmol/kg
	t-oc	Topsoil organic carbon	
	t-ece	Topsoil electric conductivity	
Soil	t-esp	Topsoil Sodicity (ESP-exchangeable sodium percentage)	%
	t-gravel	Topsoil gravel content	%
	t-sand	Topsoil sand fraction	%
	t-silt	Topsoil silt fraction	%
	t-texture	Topsoil soil texture	
	t-usda-tex-class	Topsoil USDA texture classification	
Land cover	gm-lc-v3	Land Cover Types (Cropland, Forest, Grassland, Shrubland, Wetland, Water, Tundra, Impervious Surface, Bare Land, Snow/Ice - Disturbed)	
	elev	Elevation	m
Topographical	slope		°
	aspect		rad
