# Supplementary material for: Comparative Assessment of Habitat Suitability and Niche Overlap of Three Cytospora Species in China
Source: J Fungi (Basel). 2024 Jan 3;10(1):38. doi: 10.3390/jof10010038 (PMC10817479; doi:10.3390/jof10010038)
Supplement: Supplementary file 1 [file jof-10-00038-s001.zip › Table S3.pdf]

Table S3. List of selected environmental variables for each species

Type	Code	Description	Unit	Percentage Contribution		
				C. chrysosperma	*C. mali*	*C. nivea*
Climate	bio1	Annual mean temperature	℃	64.4		
	bio2	Mean diurnal range (Mean of monthly (max.temp.-min.temp.)	℃	7.9	4.5	7.2
	bio4	Temperature seasonality (standard deviation*100)			9.5	
	bio9	Mean temperature of the driest quarter	℃		35.4	
	bio11	Mean temperature of the coldest quarter	℃			67.7
	bio13	Precipitation of the wettest month	mm	1.3		9.2
	bio14	Precipitation of the driest month	mm			3.4
	bio15	Precipitation seasonality (Coefficient of variation)		7.9	5.5	
	bio17	Precipitation of the driest quarter	mm	2.3		
	bio18	Precipitation of the warmest quarter	mm		11.3	
	bio19	Precipitation of coldest quarter	mm	0.2	18.8	1.2
Soil	t-ph	Topsoil pH (H_2O)			0.2	
	t-teb	Topsoil TEB	cmol/k			5.4
	t-esp	Topsoil Sodicity (ESP-exchangeable sodium percentage)	%			0.6
	t-gravel	Topsoil gravel content	%	7.6	3.8	
	t-texture	Topsoil soil texture				0.2
Land cover	gm-lc-v3	Land Cover Types (Cropland, Forest, Grassland,Shrubland, Wetland, Water, Tundra, Impervious Surface, Bare Land, Snow/Ice-Disturbed)		8.6	11	2.8
Topographical	elev	Elevation	m			0.9
	slope		°			1.4
