# Supplementary material for: Comparative Assessment of Habitat Suitability and Niche Overlap of Three Cytospora Species in China
Source: J Fungi (Basel). 2024 Jan 3;10(1):38. doi: 10.3390/jof10010038 (PMC10817479; doi:10.3390/jof10010038)
Supplement: Supplementary file 1 [file jof-10-00038-s001.zip › Table S4.pdf]

Table S4. Key climatic factors influencing habitat distribution of three species

Species	Environmental variables	Suitable range	Optimum	Maximum probability of existence
C.chrysosperma	Bio1(°C)	2.76–17.19	9.94	0.63
	gm_lc_v3	＞3.18	21.9	0.62
	Bio2(°C)	＞1.9	21.84	0.71
	Bio15（mm）	0–120.14	49.37	0.54
C.mali	Bio9(°C)	-10.46–9.17	-0.92	0.62
	Bio19（mm）	0–46.55	16.62	0.68
	Bio18(mm）	8.57–927.25	272.72	0.7
	gm_lc_v3	7.08–19.23	18.48	0.96
C.nivea	Bio11(°C)	-13.64–4.75	-2.09	0.64
	Bio13(mm)	0–154.09	7.52	0.66
	t_teb	0–46.58	4.25	0.65
