# Supplementary material for: Comparative Assessment of Habitat Suitability and Niche Overlap of Three Cytospora Species in China
Source: J Fungi (Basel). 2024 Jan 3;10(1):38. doi: 10.3390/jof10010038 (PMC10817479; doi:10.3390/jof10010038)
Supplement: Supplementary file 1 [file jof-10-00038-s001.zip › Table S5.pdf]

Table S5. Dynamics of changes in distribution area of *C. chrysosperma* under different climate scenariosarios

Climate scenarios		Not suitable area/%	Low suitable area/%	Moderately suitable area/%	Highly suitable area/%	Total Suitable area/%
Current climate		62.26	9.54	12.43	15.78	37.75
2021-2040	SSP126	59.36	8.74	15.75	16.16	40.65
	SSP370	62.42	9.18	13.21	15.19	37.58
	SSP585	57.52	9.4	14.88	18.2	42.48
2041–2060	SSP126	61.36	8.15	13.72	16.77	38.64
	SSP370	58.37	9.85	16.36	15.42	41.63
	SSP585	59.63	9.37	14.16	16.84	40.37
2061–2080	SSP126	64.11	8.88	12.66	14.35	35.89
	SSP370	55.49	10.37	18.47	15.67	44.51
	SSP585	60.11	9.45	17.41	13.03	39.89
2081–2100	SSP126	64.14	7.94	12.71	15.2	35.85
	SSP370	57.98	12.14	19.82	10.07	42.03
	SSP585	62.3	9.06	18.16	10.48	37.7
Mean		60.39	9.39	15.36	14.86	39.61
