# Supplementary material for: Comparative Assessment of Habitat Suitability and Niche Overlap of Three Cytospora Species in China
Source: J Fungi (Basel). 2024 Jan 3;10(1):38. doi: 10.3390/jof10010038 (PMC10817479; doi:10.3390/jof10010038)
Supplement: Supplementary file 1 [file jof-10-00038-s001.zip › Table S6.pdf]

**Table S6. Dynamics of changes in distribution area of *C. mali* under different climate scenariosarios**

| Climate scenarios | | Not suitable area/% | Low suitable area/% | Moderately suitable area/% | Highly suitable area/% | Total suitable area/% |
|---|---|---|---|---|---|---|
| Current climate | | 66.21 | 25.49 | 5.32 | 2.98 | 33.79 |
| | SSP126 | 58.94 | 30.38 | 5.79 | 4.88 | 41.05 |
| 2021-2040 | SSP370 | 59.34 | 29.83 | 6.72 | 4.11 | 40.66 |
| | SSP585 | 59.81 | 28.66 | 6.47 | 5.06 | 40.19 |
| | SSP126 | 60.38 | 27.99 | 8.17 | 3.45 | 39.61 |
| 2041–2060 | SSP370 | 56.99 | 30.89 | 7.56 | 4.56 | 43.01 |
| | SSP585 | 54.5 | 31.78 | 8.28 | 5.45 | 45.51 |
| | SSP126 | 61.29 | 27.92 | 6.06 | 4.73 | 38.71 |
| 2061–2080 | SSP370 | 53.33 | 30.94 | 10.47 | 5.26 | 46.67 |
| | SSP585 | 51.28 | 34.06 | 9.37 | 5.29 | 48.72 |
| | SSP126 | 60.03 | 29.14 | 5.82 | 5.01 | 39.97 |
| 2081–2100 | SSP370 | 45.64 | 37.14 | 11.23 | 5.98 | 54.35 |
| | SSP585 | 46.98 | 35.77 | 11.09 | 6.16 | 53.02 |
| Mean | | 56.52 | 30.77 | 7.87 | 4.84 | 25.00 |
