# Supplementary material for: Comparative Assessment of Habitat Suitability and Niche Overlap of Three Cytospora Species in China
Source: J Fungi (Basel). 2024 Jan 3;10(1):38. doi: 10.3390/jof10010038 (PMC10817479; doi:10.3390/jof10010038)
Supplement: Supplementary file 1 [file jof-10-00038-s001.zip › Table S7.pdf]

Table S7. Dynamics of changes in distribution area of *C. nivea* under different climate scenariosarios

Climate scenarios		Not suitable area/%	Low suitable area/%	Moderately suitable area/%	Highly suitable area/%	Total suitable area/%
Current climate		70.87	22.03	5.46	1.64	29.13
	SSP126	68.83	22.83	6.76	1.58	31.17
2021-2040	SSP370	70.39	22.71	5.72	1.17	29.6
	SSP585	66.48	22.81	8.45	2.26	33.52
	SSP126	67.61	21.67	8.1	2.62	32.39
2041–2060	SSP370	67.02	22.47	7.95	2.57	32.99
	SSP585	70.79	19.77	6.93	2.5	29.2
	SSP126	68.64	22.39	6.97	2	31.36
2061–2080	SSP370	59.13	28.71	9.78	2.39	40.88
	SSP585	62.94	26.39	8.63	2.03	37.05
	SSP126	70.2	21.71	6.24	2.39	30.34
2081–2100	SSP370	58.16	27.03	10.65	4.16	41.84
	SSP585	61.42	26.61	8.81	3.16	38.58
Mean		66.34	23.63	7.73	2.34	33.70
