# Supplementary material for: Comparative Assessment of Habitat Suitability and Niche Overlap of Three Cytospora Species in China
Source: J Fungi (Basel). 2024 Jan 3;10(1):38. doi: 10.3390/jof10010038 (PMC10817479; doi:10.3390/jof10010038)
Supplement: Supplementary file 1 [file jof-10-00038-s001.zip › Table S8.pdf]

Table S8. Percentage of distrbution changes of *C. chrysosperma* between current and future climate-change scenarios

Scenario	Period	Extended area（$\times 10^4$/km^2)	Shrink area（$\times 10^4$/km^2)	Stable area（$\times 10^4$/km^2)
	Current			
SSP126	2030S	32.129	12.553	185.448
	2050S	35.598	24.168	173.832
	2070S	19.326	30.789	167.211
	2090S	18.761	23.302	174.699
SSP370	2030S	20.018	21.857	176.143
	2050S	38.092	22.668	175.332
	2070S	64.109	36.904	161.097
	2090S	56.929	73.397	124.604
SSP585	2030S	41.112	9.391	188.609
	2050S	44.675	25.209	172.791
	2070S	47.855	50.257	147.743
	2090S	57.104	76.559	121.442
