# Supplementary material for: Comparative Assessment of Habitat Suitability and Niche Overlap of Three Cytospora Species in China
Source: J Fungi (Basel). 2024 Jan 3;10(1):38. doi: 10.3390/jof10010038 (PMC10817479; doi:10.3390/jof10010038)
Supplement: Supplementary file 1 [file jof-10-00038-s001.zip › Table S9.pdf]

Table S9. Percentage of distrbution changes of *C.mali* between current and future climate-change scenarios

Scenario	Period	Extended area ($\times10^4$/km^2)	Shrink area ($\times10^4$/km^2)	Stable area ($\times10^4$/km^2)
Current				
SSP126	2030S	21.274	1.477	50.168
	2050S	21.299	7.069	44.576
	2070S	23.324	2.962	48.684
	2090S	23.451	4.107	47.538
SSP370	2030S	2.441	1.168	50.477
	2050S	27.495	4.087	47.558
	2070S	49.373	10.082	41.563
	2090S	57.983	9.268	42.377
SSP585	2030S	27.014	1.003	50.642
	2050S	36.954	6.238	45.407
	2070S	45.572	9.945	41.7
	2090S	61.746	11.324	40.322
