# Supplementary material for: Comparative Assessment of Habitat Suitability and Niche Overlap of Three Cytospora Species in China
Source: J Fungi (Basel). 2024 Jan 3;10(1):38. doi: 10.3390/jof10010038 (PMC10817479; doi:10.3390/jof10010038)
Supplement: Supplementary file 1 [file jof-10-00038-s001.zip › TITLE NAME.pdf]

Figure 1. Global Occurrence records of three studied species. The pink, green and black dots represent *C. chrysosperma*, *C. mali* and *C. nivea*, respectively.

Figure 2. The receiver operating characteristic curve for target species. A, B and C represent *C. chrysosperma*, *C. mali* and *C. nivea*, respectively.

Figure 3. The current potentially geographical distribution for three studied species in China. A, B and C represent *C. chrysosperma* (no suitability $p \leq 0.2812$; low suitability $0.2812 < p \leq 0.4$; medium suitability $0.4 < p \leq 0.6$; high suitability $p > 0.6$, p = probability), *C. mali* (no suitability $p \leq 0.0887$; low suitability $0.0887 < p \leq 0.4$; medium suitability $0.4 < p \leq 0.6$; high suitability $p > 0.6$, p = probability) and *C. nivea* (no suitability $p \leq 0.1184$; low suitability $0.1184 < p \leq 0.4$; medium suitability $0.4 < p \leq 0.6$; high suitability $p > 0.6$, p = probability), respectively.

Figure 4. Centroid shifts of potential suitable area for three studied species under different climatic scenarios in China. Red star indicates the centroids of the suitable habitats of each species under current climate. Dots and triangles represent the centroids of the suitable habitats of each species under different future climate scenarios. A, B and C represent *C. chrysosperma*, *C. mali* and *C. nivea*, respectively.

Figure 5. Niche and range overlap of the potential distribution habitat of the three species.

Table 1. Results of MaxEnt models with optimized parameters developed for three studied species.

Table 2. Centroid migration of suitable areas under future climatic conditions for target species

Table 3. Niche and range overlap of the potential distribution habitat of the three species

SUPPLEMENTARY MATERIALS

Figure S1 Correlation analysis of various environmental factors

Figure S2 Jackknife test of variable importance Regularized training gain

Figure S3 Response curves for predictiors in MaxEne model

Figure S4 The future potentially geographical distributions of C. chrysosperma in China under future climatic conditions

Figure S5 The future potentially geographical distributions of C. mali in China under future climatic conditions

Figure S6 The future potentially geographical distributions of C. nivea in China under future climatic conditions

Figure S7 Adaptability changes in C. chrysosperma under different future climate scenarios

Figure S8 Adaptability changes in C. mali under different future climate scenarios

Figure S9 Adaptability changes in C. nivea under different future climate scenarios

Table S1 Geographical distributions of C. chrysosperma , C.mali and C.nivea species sampled in this study

Table S2 Environmental variables for current period model analysis

Table S3 List of selected environmental variables for each species

Table S4 Key climatic factors influencing habitat distribution of three species

Table S5 Dynamics of changes in distribution area of C. chrysosperma under different climate scenariosarios

Table S6 Dynamics of changes in distribution area of C. mali under different climate scenariosarios

Table S7 Dynamics of changes in distribution area of C. nivea under different climate scenariosarios

Table S8 Percentage of distrbution changes of C. chrysosperma between current and future climate-change scenarios

Table S9 Percentage of distrbution changes of C.mali between current and future climate-change scenarios

Table S10 Percentage of distrbution changes of C. nivea between current and future climate-change scenarios
