# Supplementary figures and images for: Comparative Assessment of Habitat Suitability and Niche Overlap of Three Cytospora Species in China
Source: J Fungi (Basel). 2024 Jan 3;10(1):38. doi: 10.3390/jof10010038 (PMC10817479; doi:10.3390/jof10010038)

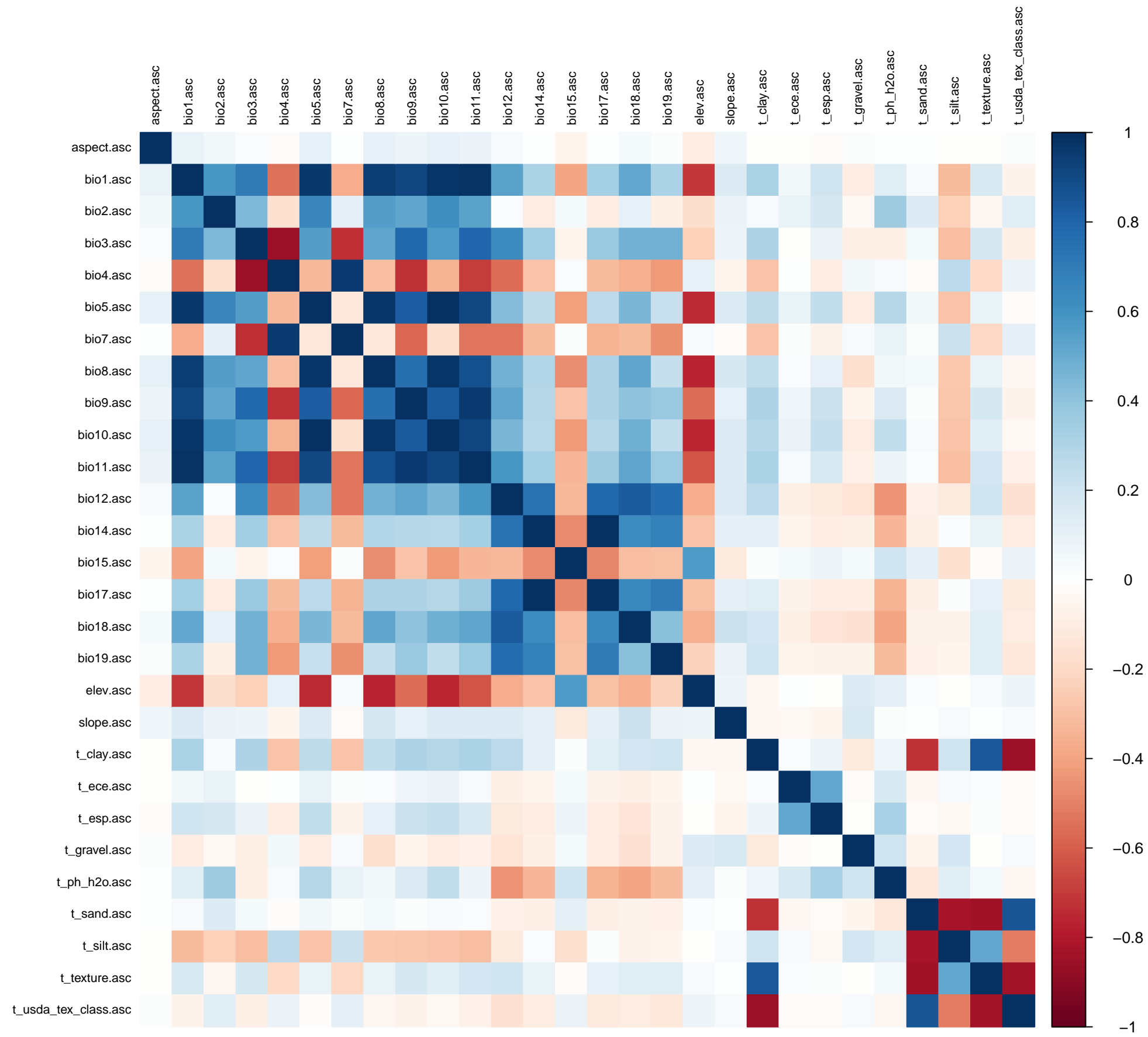

Supplement: Supplementary file 1 [file jof-10-00038-s001.zip › Figure S1.pdf]

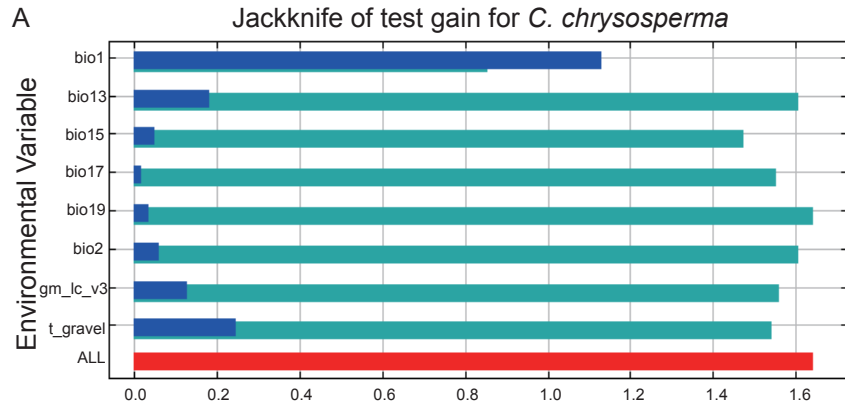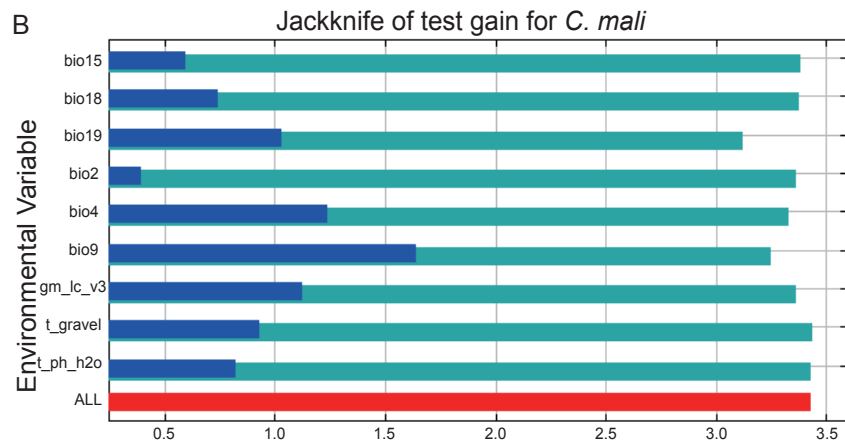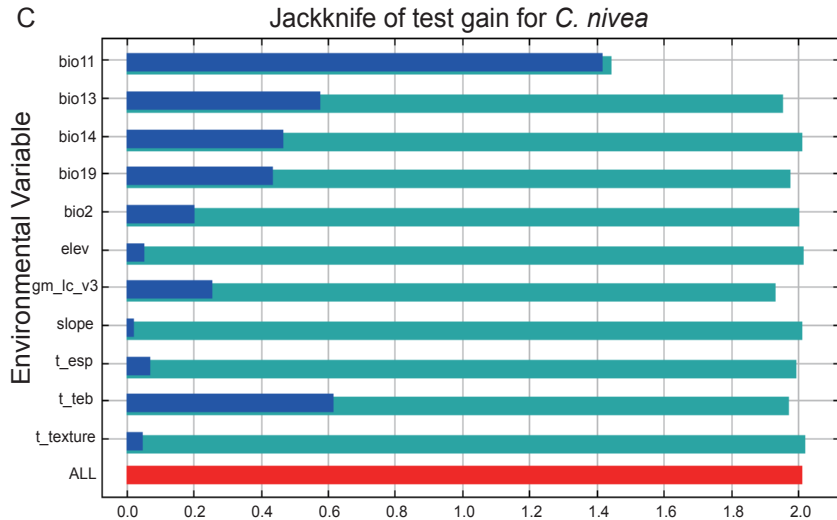

Without variable

With only variable

With all variable

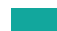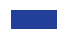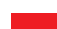

Supplement: Supplementary file 1 [file jof-10-00038-s001.zip › Figure S2.pdf]

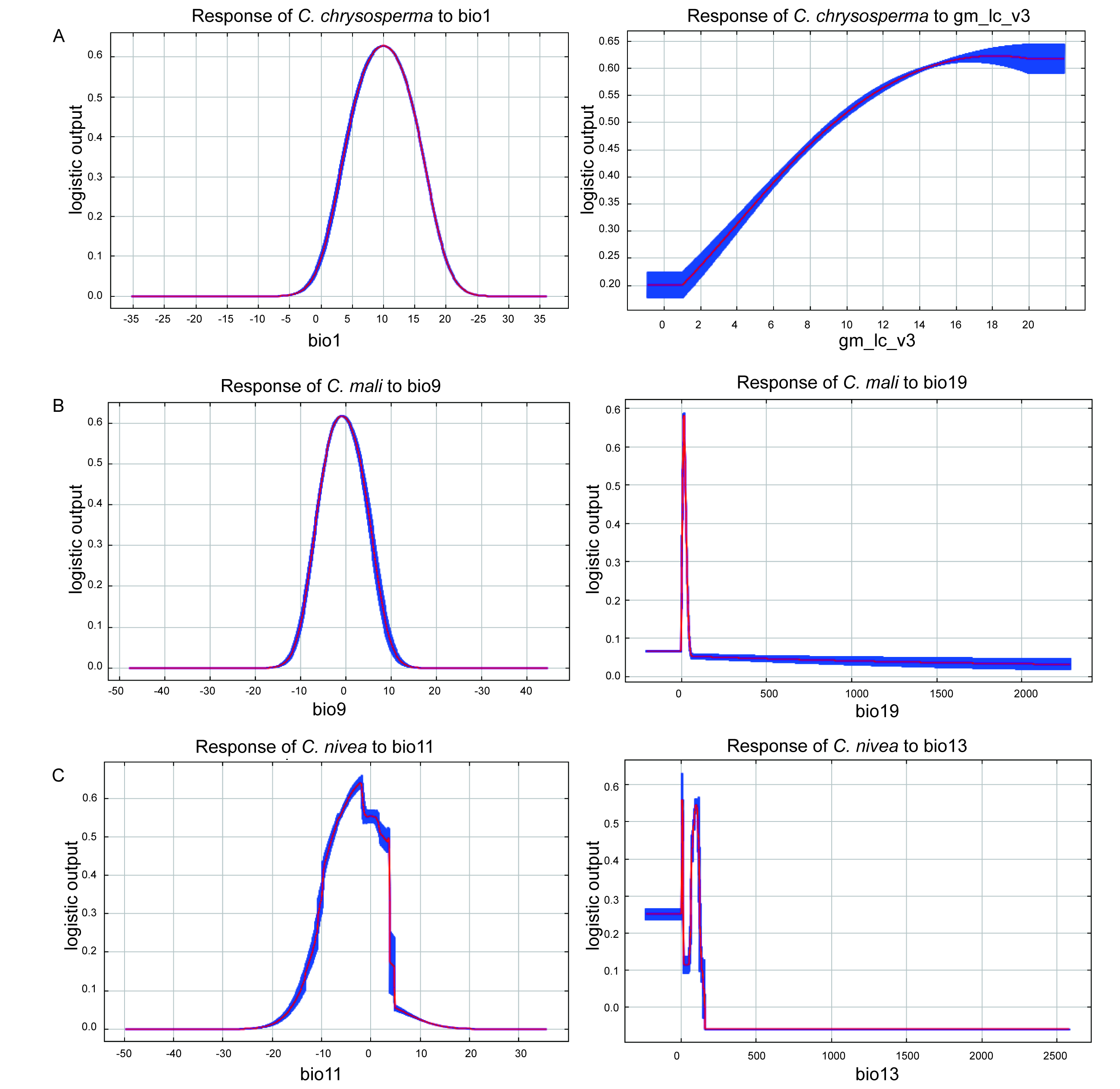

Supplement: Supplementary file 1 [file jof-10-00038-s001.zip › Figure S3.tif]

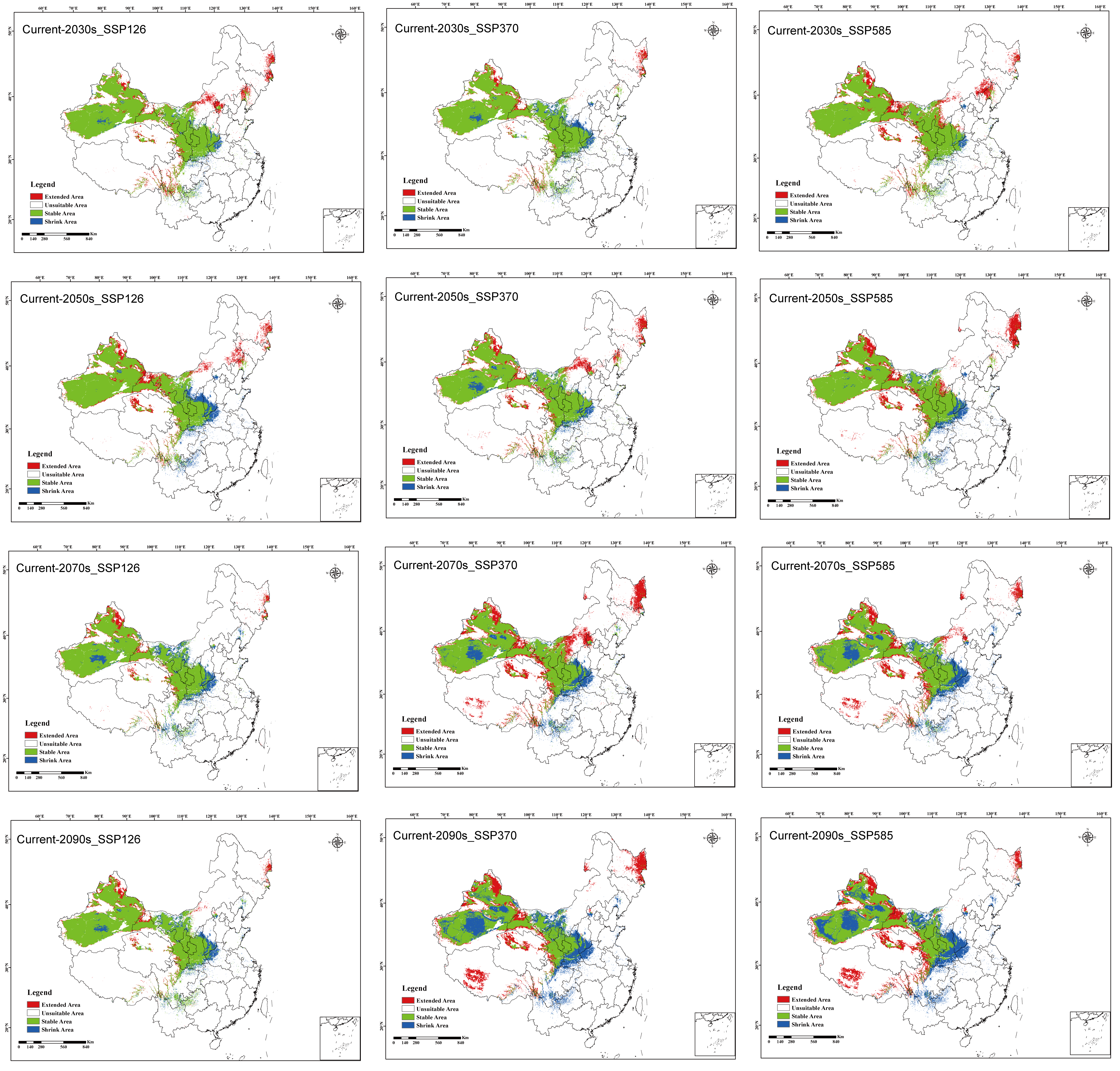

Supplement: Supplementary file 1 [file jof-10-00038-s001.zip › Figure S7.tif]

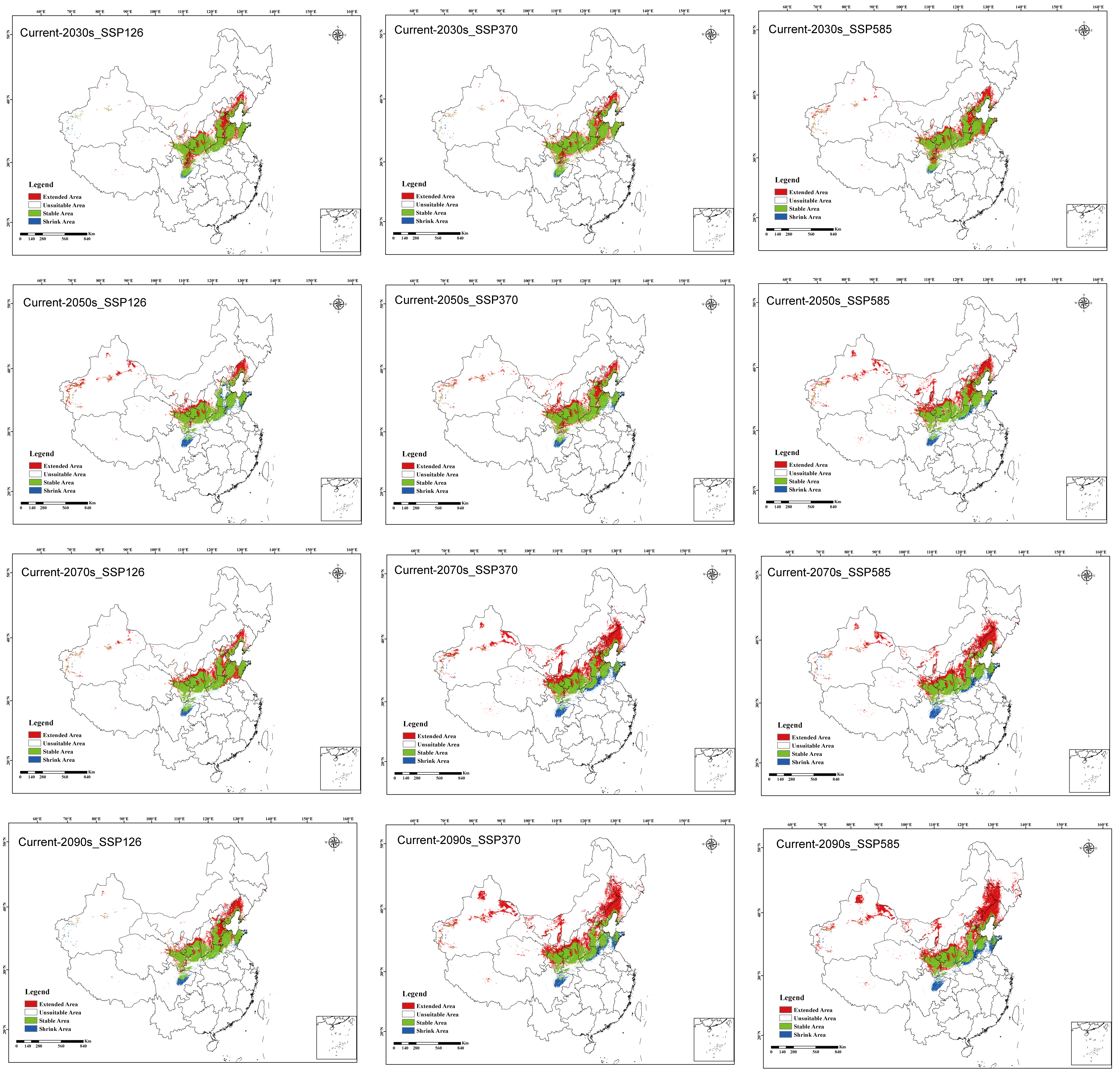

Supplement: Supplementary file 1 [file jof-10-00038-s001.zip › Figure S8.tif]
